# Iron-Catalyzed Synthesis, Structure, and Photophysical Properties of Tetraarylnaphthidines

**DOI:** 10.3390/molecules25071608

**Published:** 2020-04-01

**Authors:** Alexander Purtsas, Sergej Stipurin, Olga Kataeva, Hans-Joachim Knölker

**Affiliations:** 1 Faculty of Chemistry, Technische Universität Dresden, Bergstraße 66, 01069 Dresden, Germany; alexander.purtsas@chemie.tu-dresden.de (A.P.); sergej.stipurin@chemie.tu-dresden.de (S.S.); 2 A. E. Arbuzov Institute of Organic and Physical Chemistry, FRC Kazan Scientific Center, Russian Academy of Sciences, Arbuzov Str. 8, 420088 Kazan, Russia; olga-kataeva@yandex.ru

**Keywords:** iron catalysis, oxidative coupling, naphthidines, fluorescence

## Abstract

We describe the synthesis and photophysical properties of tetraarylnaphthidines. Our synthetic approach is based on an iron-catalyzed oxidative C–C coupling reaction as the key step using a hexadecafluorinated iron–phthalocyanine complex as a catalyst and air as the sole oxidant. The *N*,*N*,*N*’,*N*’-tetraarylnaphthidines proved to be highly fluorescent with quantum yields of up to 68%.

## 1. Introduction

Triarylamines have been the subject of numerous studies due to their electron-rich character and the resulting low oxidation potentials [1,2,3]. These unique properties of triarylamine derivatives have induced extensive investigations towards their potential applications in organic films for optoelectronics [4] and as hole-transport materials in OLEDs (organic light emitting diodes) and solar cells [5,6,7,8]. Naphthidines (1,1′-binaphthalene-4,4′-diamines) have also been studied because of their ready oxidation to radical cations [9,10,11,12]. Starting from the corresponding 1-naphthylamines by an oxidative coupling, the synthesis of naphthidines can be induced electrochemically [9,10,13], by stoichiometric amounts of titanium tetrachloride [11], stoichiometric amounts of iron(III) chloride [14], chloranil [15], or (bis(trifluoroacetoxy)iodo)benzene (PIFA) [16]. It has been well-known for a long time that naphthidines can be separated into their stable atropisomers [17].

**Figure 1:**
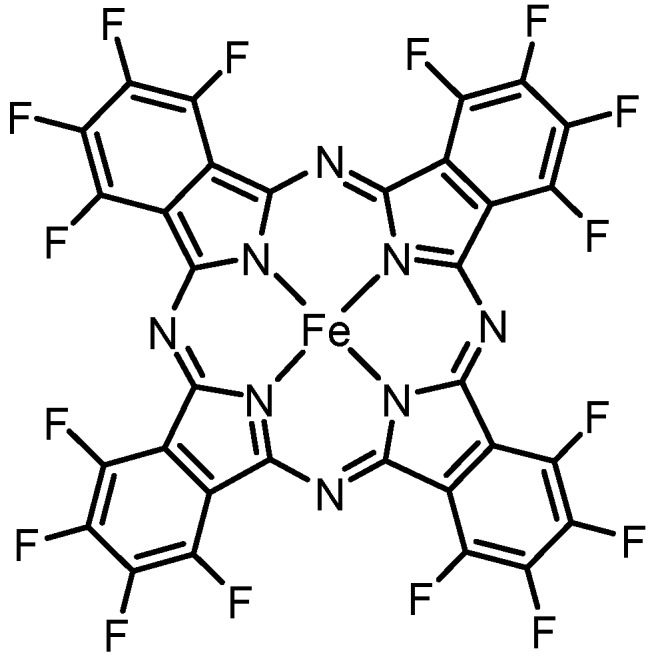
Structural formula of iron(II)–hexadecafluorophthalocyanine (FePcF_16_).

Herein, we report a simple synthesis of *N*,*N*,*N*’,*N*’-tetraarylnaphthidines by an iron-catalyzed oxidative coupling of the corresponding 1-(diphenylamino)naphthalene precursor using iron(II)–hexadecafluorophthalocyanine (FePcF_16_) [18] as the catalyst and air as the final oxidant (Figure 1). Moreover, we have investigated the photophysical properties of the obtained naphthidine derivatives.

## 2. Results and Discussion

### 2.1. Synthesis

**Scheme 1:**
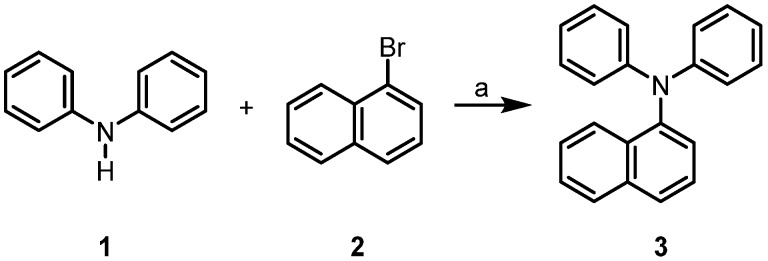
Synthesis of 1-(diphenylamino)naphthalene (**3**). *Reagents and conditions:* (**a**) 1 mol% Pd(OAc)_2_, 2 mol% RuPhos, 1.2 equivalents NaO*t*Bu, air, 110 °C, 12 h, 89% compound **3**.

Following a procedure reported by Nechaev et al. for the Buchwald–Hartwig reaction under solvent-free conditions [19], we have achieved the coupling of diphenylamine (**1**) and 1-bromonaphthalene (**2**) to afford 1-(diphenylamino)naphthalene (**3**) in high yield (Scheme 1).

**Scheme 2:**
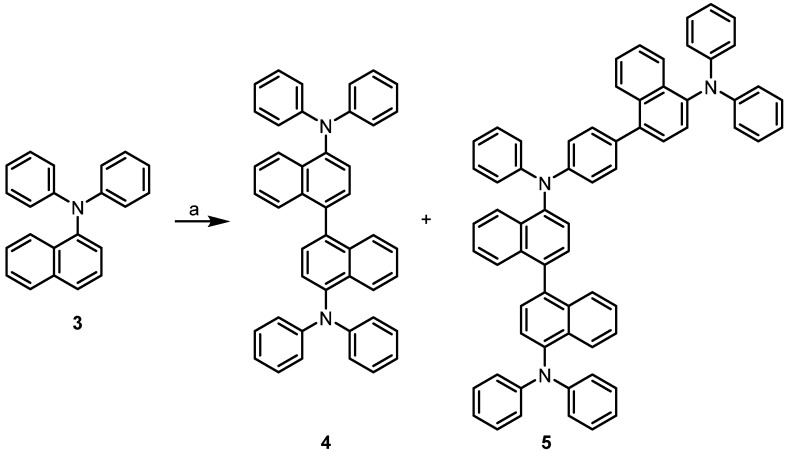
Iron-catalyzed oxidative C–C coupling of 1-(diphenylamino)naphthalene (**3**). *Reagents and conditions:* (**a**) 2 mol% FePcF_16_, 40 mol% MsOH, CH_2_Cl_2_, air, room temperature, 24 h, 48% compound **4**, 11% compound **5**.

Then, we turned our attention to the projected iron-catalyzed oxidative homocoupling of 1-(diphenylamino)naphthalene (**3**). Recently, we applied our iron-catalyzed oxidative coupling methodology to the C–C homocoupling of diarylamines, 1-, and 2-hydroxycarbazoles, as well as to the cross coupling of tertiary anilines with hydroxyarenes [20,21,22]. Iron as a first-row transition metal is environmentally safe and has become a powerful tool in synthetic organic chemistry with many applications for selective C–H bond activation [23,24,25,26]. According to a previous report by Yang, *N*,*N*,*N*’,*N*’-tetraphenylnaphthidine (**4**) could not be prepared by oxidative coupling of compound **3** with stoichiometric amounts of iron(III) chloride [14]. The oxidation of compound **3** with chloranil provides compound **4** only as the minor isomer in an inseparable mixture with the corresponding benzidine derivative [15]. Based on our previous studies [20,21,22], we envisaged to develop an iron-catalyzed homocoupling of the triarylamine **3** with air as the sole oxidant. Using catalytic amounts (2 mol%) of FePcF_16_ and substoichiometric amounts (40 mol%) of methanesulfonic acid as an additive at room temperature provided *N*,*N*,*N*’,*N*’-tetraphenylnaphthidine (**4**) in 48% yield along with the *N*,*N*,*N*’,*N*’-tetraarylnaphthidine **5** as a by-product in 11% yield (Scheme 2). Obviously, compound **5** results from a further oxidative C–C coupling at the *p*-position of one of the phenyl rings of the initial coupling product compound **4**. The structural assignments for compounds **4** and **5** were based on their ^1^H-NMR and ^13^C-NMR spectroscopic data and an X-ray analysis of compound **4** (see Section 2.2).

### 2.2. Structure

**Figure 2:**
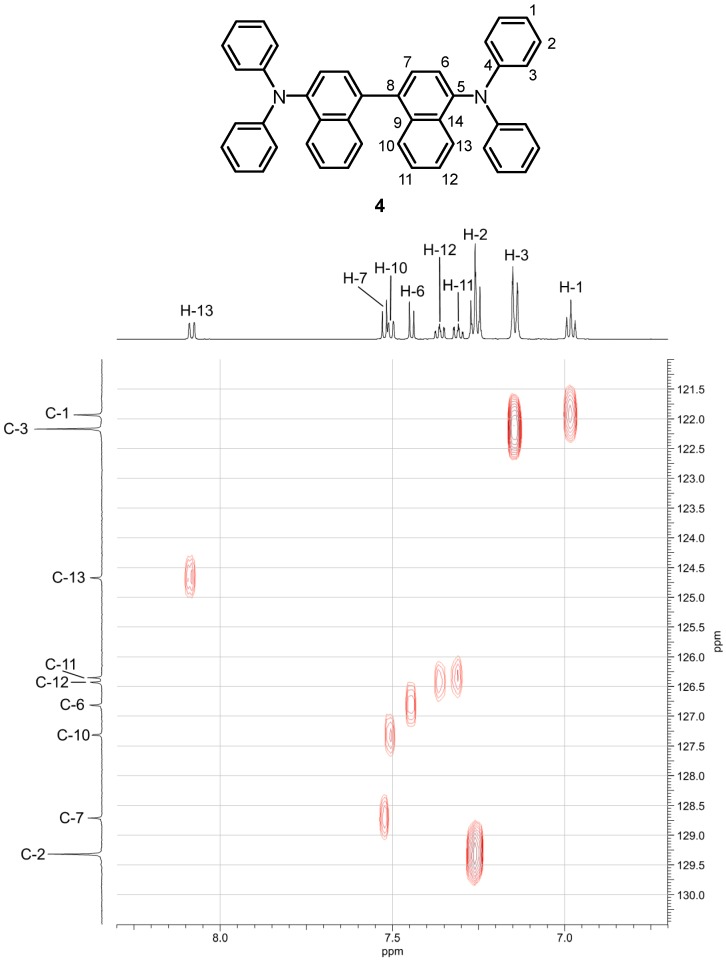
Assignment of the ^13^C-NMR signals of the *N*,*N*,*N*’,*N*’-tetraphenylnaphthidine (**4**) to the respective ^1^H-NMR signals by the HSQC spectrum.

The ^1^H-NMR, ^13^C-NMR, and DEPT spectra of compound **4** displayed signals for a highly symmetrical compound. Signals for nine aromatic CH and five quaternary aromatic carbon atoms were identified. The COSY experiment revealed the presence of three spin systems (Supplementary Materials, COSY of compound **4**). The first is caused by coupling of H-1 with H-2 and H-3, which belong to the phenyl fragment. The second spin system consists of H-6 and H-7. The third system is formed by H-10, H-11, H-12, and H-13. The assignment of all ^13^C-NMR signals to the respective ^1^H-NMR signals could be achieved by an HSQC measurement (Figure 2 and Table S1). The constitution of naphthidine **4** was confirmed by analysis of the HMBC spectrum (Supplementary Materials, HMBC of compound **4**, Table S1). Characteristic HMBC signals (C-4/H-2 and C-4/H-3) led to elucidation of the quaternary carbon atom C-4 by connecting the proton spin systems. The position of C-5 was established based on the HMBC cross-peaks with H-6, H-7, and H-13. Accordingly, the quaternary aromatic carbon atom C-8 was assigned based on the HMBC interactions with H-6, H-7, and H-10. HMBC cross-peaks between C-9/H-7, H-11, H-13 and C-14/H-6, H-10, H-12 unambiguously clarified the location of both naphthyl-based quaternary carbon atoms. The NOE interactions were exploited to confirm the positions of H-3 and H-13 (Supplementary Materials, NOESY of compound **4**). Due to the interactions of these fjord region protons, H-13 showed a substantial downfield shift to 8.08 ppm. Moreover, H-2, H-3, and H-6 exhibited an interaction with the ^15^N atom (Supplementary Materials, ^1^H/^15^N HMBC of compound **4**).

**Figure 3:**
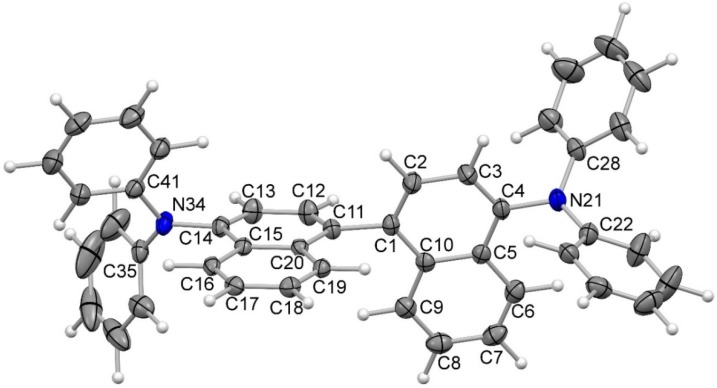
Molecular structure of *N*,*N*,*N*’,*N*’-tetraphenylnaphthidine (**4**) in the crystal (ORTEP plot showing thermal ellipsoids at the 50% probability level). Selected bond lengths (Å): C1–C2 1.370(3), C1–C10 1.424(2), C5–C10 1.431(2), C4–N21 1.436(2), C1–C11 1.500(2), C11–C12 1.370(2), C11–C20 1.428(2), C15–C20 1.432(2), C14–N34 1.430(2). Selected bond angles (°): C10–C1–C11 121.4(2), C2–C1–C11 118.8(2), C2–C1–C10 119.7(2), C1–C10–C9 122.2(2), C3–C4–N21 120.6(2), C5–C4–N21 120.0(2), C4–N21–C22 117.1(2), C4–N21–C28 118.3(2), and C22–N21–C28 120.6(2).

**Figure 4:**
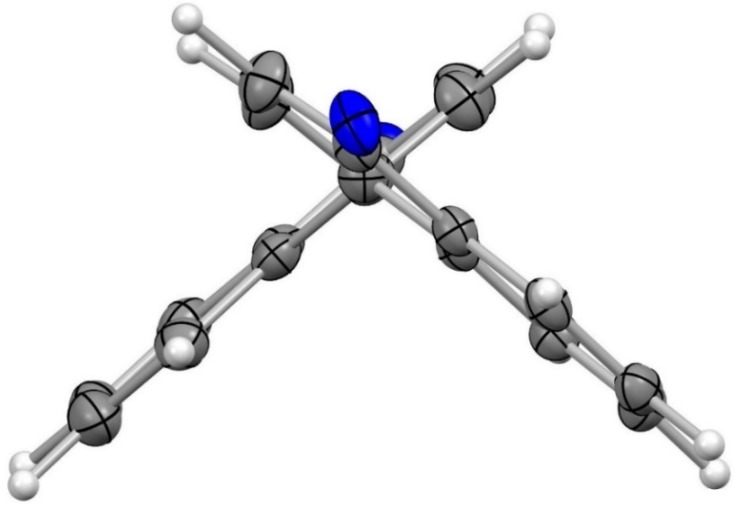
Crystal structure of compound **4** with a view along the N21–C4/C1–C11/C14–N34 axis (ORTEP plot showing thermal ellipsoids at the 50% probability level, the *N*–phenyl substituents have been omitted for clarity). Torsional angle (C2–C1–C11–C12) 99.4(2)°.

**Figure 5:**
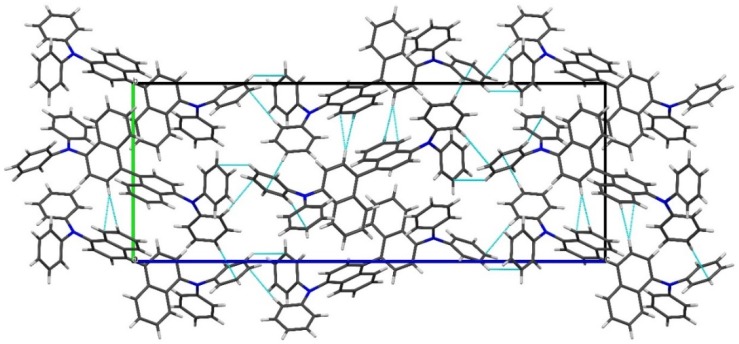
Fragment of the crystal packing of *N*,*N*,*N*’,*N*’-tetraphenylnaphthidine (**4**) showing the unit cell and C–H···π interactions (view along the *a*-axis of the crystal).

The structural assignment for *N*,*N*,*N*’,*N*’-tetraphenylnaphthidine (**4**) was confirmed by an X-ray crystal structure determination (Figure 3). The molecule of compound **4** adopts a *trans* conformation around the central biaryl bond (C1–C11) with a torsional angle of 99.4(2)° (Figure 4) according to the notation accepted for binaphthyl derivatives [27,28]. The geometry of the naphtho fragments exhibiting large variations of the bond lengths is in agreement with the crystal structure of naphthalene [29,30] and its structure obtained by quantum chemical calculations [31]. The orientation of the phenyl groups attached to the nitrogen atoms at the two sides of the molecule is different. The two phenyl rings at N21 are symmetrically oriented respective to the bisector plane drawn through the C4–N21 bond, while the two phenyl groups at N34 have different orientations. In the crystal, the molecules of compound **4** participate in multiple weak intermolecular C–H···π interactions between the naphtho groups (Figure 5). The distances between the hydrogen atoms and the sp^2^-carbon atoms range from 2.7 to 2.8 Å and the angles are typical for these weak hydrogen bonds [32]. This sort of packing with face-to-edge interactions has been observed also in naphthalene itself [29,30] and in binaphthyl derivatives [27,28]. It is noteworthy that the naphtho groups interact mostly with each other and the phenyl groups interact predominantly with phenyl groups, whereas naphtho–phenyl contacts are rare.

**Figure 6:**
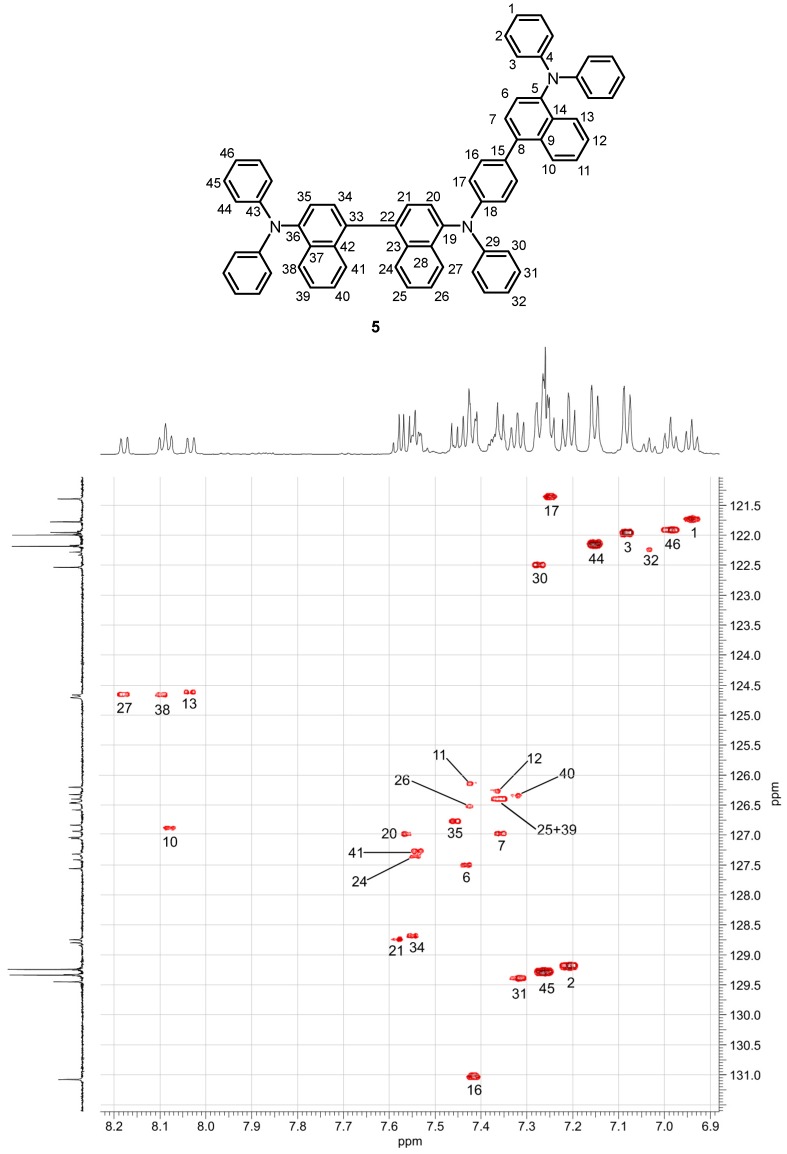
Assignment of the ^13^C-NMR signals of the *N*,*N*,*N*’,*N*’-tetraarylnaphthidine (**5**) to the respective ^1^H-NMR signals by the HSQC spectrum.

The connectivity in compound **5** has been secured by a full set of 2D-NMR spectra (see below and the copies of the 2D-NMR spectra in the Supplementary Materials). The NMR spectra of **5** display signals for a non-symmetrical aromatic compound. The JRES experiment revealed the absence of a singlet. Thus, the additional oxidative coupling of the naphthidine **4** with compound **3** did not occur at positions 2, 6, 7, 11, or 12 (Supplementary Materials, JRES of compound **5**). The ^1^H-NMR spectrum in combination with the HSQC (Figure 6) and the JRES spectra displayed signals for four substantially downfield-shifted doublets. On the basis of the fjord region effect observed for compound **4**, these four downfield-shifted signals in the ^1^H-NMR spectrum of compound **5** are the naphthyl protons H-10, H-13, H-27, and H-38. The signals of the four corresponding carbon atoms are detected at δ_C_ = 124.66 (C-13), 124.70 (C-27), 124.71 (C-38), and 126.93 (C-10) ppm in the ^13^C-NMR spectrum (HSQC in Figure 6, Table S2). The NOE interactions indicated the positions of the protons at the phenyl groups (Supplementary Materials, NOESY of compound **5**). The positions of the protons at C-3, C-30, and C-44 have been identified unambiguously by NOE interactions with H-13, H-27, and H-38. Support for this assignment of the protons H-3, H-30, and H-44 is obtained from an HMBC interaction with ^15^N (Supplementary Materials, ^1^H/^15^N HMBC of compound **5**). A strong NOE correlation of H-10 with H-16 and the presence of a spin interaction between H-16 and H-17 in the COSY (Supplementary Materials, COSY of compound **5**) confirmed an oxidative coupling of compound **3** at C-1 of naphthidine **4**. Further support was obtained from HMBC correlations of H-16 with the quaternary carbon atoms C-8 at δ_C_ = 138.64 ppm and C-18 at δ_C_ = 147.93 ppm and the HMBC correlation of H-17 with C-15 at δ_C_ = 133.51 ppm (Supplementary Materials, HMBC of compound **5**). The obtained NMR data are in excellent agreement with the structure assigned for compound **5**.

### 2.3. Photophysical Studies

**Figure 7:**
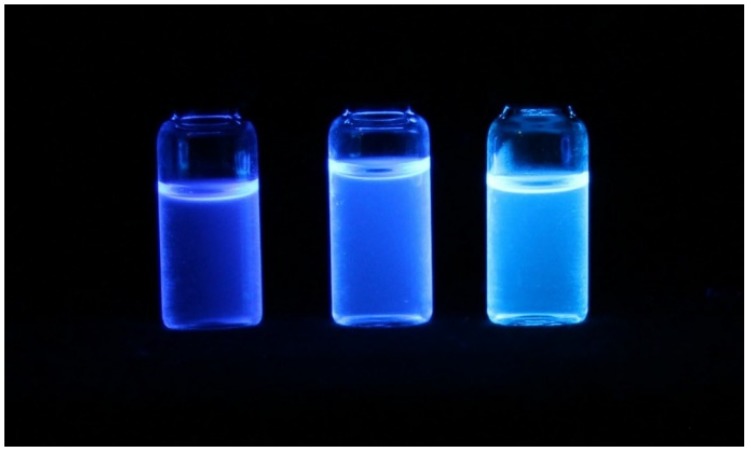
Fluorescence of the compounds **3** (left), **4** (center), and **5** (right) in methanol (concentration *c* = 2 mg mL^−1^) by excitation with UV light (λ_ex_ = 254 nm).

**Figure 8:**
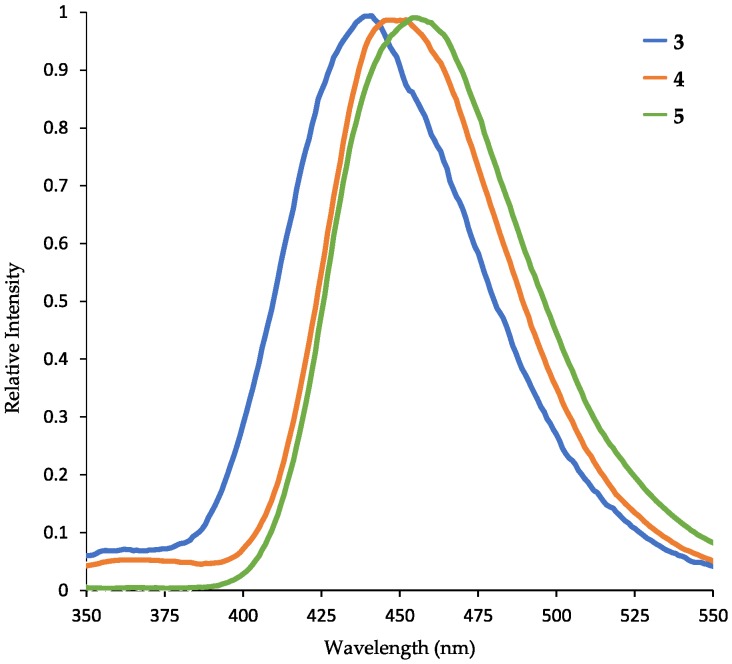
Normalized fluorescence emission spectra of 1-(diphenylamino)naphthalene (**3**) (blue), *N*,*N*,*N*’,*N*’-tetraphenylnaphthidine (**4**) (orange), and naphthidine **5** (green) in methanol (excitation at λ_ex_ = 291 nm).

On irradiation with UV-light (λ_ex_ = 254 nm) in methanolic solution, 1-(diphenylamino)naphthalene (**3**) and the resulting *N*,*N*,*N*’,*N*’-tetraarylnaphthidines **4** and **5** show a strong blue fluorescence (Figure 7). Therefore, we investigated the photophysical properties of compounds **3**, **4**, and **5** in more detail. The UV absorption and fluorescence emission data for all three compounds are listed in Table 1 and the normalized fluorescence emission spectra are displayed in Figure 8. The fluorophores **3**, **4**, and **5** exhibit large Stokes shifts of 149 to 162 nm. While the UV absorption maxima are identical, the fluorescence emission maxima show a bathochromic shift of only 7 nm for *N*,*N*,*N*’,*N*’-tetraphenylnaphthidine (**4**) and 14 nm for compound **5**, as compared to 1-(diphenylamino)naphthalene (**3**). Previously, even larger Stokes shifts have been observed for *N*,*N*-dimethylaminonaphthalenes [33] and strong bathochromic shifts have been reported for substituted triarylamines [34].

For the naphthidines **4** and **5**, the UV absorption and fluorescence emission maxima along with fluorescence quantum yields, fluorescence lifetimes, and CIE-coordinates were determined (Table 2).

The UV absorption maximum for the naphthidines **4** and **5** is at the same wavelength (294 nm), whereas the fluorescence emission maximum of compound **5** shows a bathochromic shift of 4 nm as compared with compound **4**. The fluorescence quantum yields of compounds **4** and **5** are relatively high and in the same range (68% and 65%, respectively, by excitation at 310 nm). Compound **5** exhibits a shorter fluorescence lifetime of 3.6 ns as compared with 4.2 ns for *N*,*N*,*N*’,*N*’-tetraphenylnaphthidine (**4**). The CIE-coordinates for the blue light emissions of compounds **4** and **5** are indicative of a blue light which could be useful for applications in OLEDs [35,36,37,38,39]. The fluorescence quantum yields are very comparable with compounds already investigated as blue fluorescent OLEDs [37,38,39]. It has been demonstrated for other naphthalene fluorophores that a bathochromic shift of the emission can be achieved by increasing the size of the π-system and by the introduction of appropriate substituents [40,41]. Thus, application of these tools should easily allow an optimization of the present fluorophoric system.

**Figure 9:**
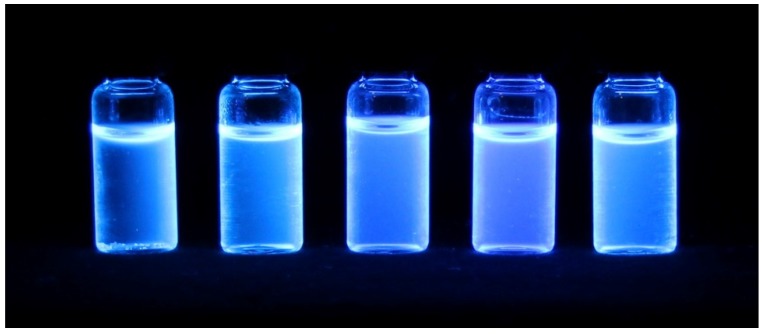
Fluorescence of *N*,*N*,*N*’,*N*’-tetraphenylnaphthidine (**4**) by excitation with UV light (λ_ex_ = 254 nm) in different solvents (concentration *c* = 2 mg mL^−1^ and from left to right, isohexane, dichloromethane, ethyl acetate, THF, and methanol).

In addition, we investigated the absorption and fluorescence properties of *N*,*N*,*N*’,*N*’-tetraphenylnaphthidine (**4**) in various nonpolar and polar solvents. The fluorescence behavior of compound **4** on excitation at 254 nm was demonstrated in isohexane, dichloromethane, ethyl acetate, THF, and methanol (Figure 9). The corresponding UV absorption and fluorescence emission spectra are shown in the Supplementary Materials. The corresponding photophysical data of compound **4** are summarized in Table 3. For a series of *N*,*N*-dimethylaminonaphthalene fluorophores, Brummond et al. observed a significant red shift of the fluorescence emission maxima by increasing the solvent polarity [33], whereas in other systems the solvent dependency of the emission was significantly lower [37]. For *N*,*N*,*N*’,*N*’-tetraphenylnaphthidine (**4**), this solvatochromic shift is much less pronounced (about 21 nm in dichloromethane as compared with the corresponding value in isohexane) (Table 3).

## 3. Materials and Methods

### 3.1. General

All reactions were performed in oven-dried glassware using anhydrous solvents under argon, unless stated otherwise. Pd(OAc)_2_ was recrystallized from glacial AcOH. All other chemicals were used as received from commercial sources. The iron(III)-catalyzed reactions were carried out in non-dried solvents under air. Iron(II)–hexadecafluorophthalocyanine was prepared following a procedure reported previously [18]. Flash chromatography was performed using silica gel from Acros Organics (0.035 to 0.070 mm). Automated flash chromatography was performed on a Büchi Sepacore system equipped with an UV monitor on silica gel (Acros Organics, 0.035 to 0.070 mm). The TLC was performed with TLC plates from Merck (60 F254) using UV light for visualization. The melting points were measured on a Gallenkamp MPD 350 melting point apparatus. Ultraviolet spectra were recorded on a PerkinElmer 25 UV/Vis spectrometer. The fluorescence spectra were measured on a Varian Cary Eclipse spectrophotometer. The IR spectra were recorded on a Thermo Nicolet Avatar 360 FT-IR spectrometer using the ATR method (attenuated total reflectance). The NMR spectra were recorded on Bruker DRX 500 and Avance III 600 spectrometers. The chemical shifts δ were reported in ppm with the solvent signal as internal standard. Standard abbreviations were used to denote the multiplicities of the signals. EI mass spectra were recorded by GC/MS-coupling using an Agilent Technologies 6890 N GC System equipped with a 5973 Mass Selective Detector (70 eV). The ESI-MS spectra were recorded on an Esquire LC using an ion trap detector from Bruker. Positive and negative ions were detected. Elemental analyses were measured on a EuroVector EuroEA3000 elemental analyzer. The X-ray crystal structure analyses were performed with a Bruker–Nonius Kappa CCD and with a Bruker AXS Smart APEX diffractometer equipped with a 700 series Cryostream low temperature device from Oxford Cryosystems. SHELXS-97 [42], SADABS version 2.10 [43], SHELXL-97 [44], POV-Ray for Windows version 3.7.0.msvc10.win64, and ORTEP-3 for Windows [45] were used as software.

### 3.2. Photoluminescence Measurements

The 2 wt% emitter films were prepared by doctor blading a solution of an emitter in a 10 wt% poly(methyl methacrylate) solution in dichloromethane on a quartz substrate with a 60 μm doctor blade. The film was dried, and the emission measured under nitrogen atmosphere. Excitation was conducted in a wavelength range of 250–400 nm (Xe lamp with monochromator), and the emission was detected with a calibrated quantum yield detection system (Hamamatsu, model C11347-01). The fluorescence lifetime was measured with an Edinburgh Instruments mini-τ by excitation with pulses of an EPLED (Edinburgh picosecond Pulsed Light Emitting Diode) (360 nm, 20 kHz) and time-resolved photon counting (TCSPC).

### 3.3. Procedures

#### 3.3.1. *N*,*N*-Diphenylnaphthalen-1-amine (1-(Diphenylamino)naphthalene) (3) 

Compound **3** was prepared following a literature procedure [19]. To a screw-cap vial were added 1-bromonaphthalene (**2**) (307 µL, 455 mg, 2.20 mmol), diphenylamine (**1**) (338 mg, 2.00 mmol), Pd(OAc)_2_ (5.4 mg, 24 µmol), RuPhos (19 mg, 41 µmol), and powdered NaO*t*Bu (236 mg, 2.46 mmol). The reaction mixture was stirred at 110 °C for 12 h. After cooling to room temperature, the mixture was dissolved in CH_2_Cl_2_/H_2_O (1:1). The aqueous layer was extracted twice with dichloromethane (5 mL). The combined organic layers were dried over MgSO_4_, the solvent was evaporated in vacuo, and the crude product was purified by chromatography on silica gel with isohexane/dichloromethane (30:1) to afford compound **3** (523 mg, 1.77 mmol, 89%) as a colorless solid. M.p. 132.5 °C; UV (MeOH) *λ* = 219, 290, and 336 nm; fluorescence (MeOH) *λ*_ex_ = 291 and *λ*_em_ = 439 nm; IR (ATR) *ν =* 3036, 1934, 1740, 1586, 1563, 1488, 1389, 1341, 1290, 1272, 1176, 1087, 1026, 1014, 891, 798, 773, 749, 693, and 624 cm^−1^; ^1^H-NMR (500 MHz, CDCl_3_) *δ* = 6.93 (t, *J* = 7.5 Hz, 2H), 7.03 (dd, *J* = 8.7, 0.8 Hz, 4H), 7.15–7.22 (m, 4H), 7.30–7.38 (m, 2H), 7.42–7.50 (m, 2H), 7.77 (d, *J* = 8.2 Hz, 1H), 7.88 (d, *J* = 8.2 Hz, 1H), and 7.94 (d, *J* = 8.5 Hz, 1H); ^13^C-NMR and DEPT at θ = 135° (125 MHz, CDCl_3_) *δ* = 121.76 (2 CH), 121.97 (4 CH), 124.41 (CH), 126.25 (CH), 126.48 (CH), 126.51 (CH), 126.56 (CH), 127.39 (CH), 128.51 (CH), 129.21 (4 CH), 131.41 (C), 135.41 (C), 143.71 (C), 148.58 (2 C); MS (EI): *m/z* (%) = 295 (100, [M]^+^), 294 (45), 293 (11), 217 (23), and 77 (10); elemental analysis calcd. for C_22_H_17_N, C 89.46, H 5.80, and N 4.74; found, C 89.61, H 6.05, and N 4.76.

#### 3.3.2. Iron-Catalyzed Oxidative C−C Coupling 

Dichloromethane (9 mL) was added to a mixture of iron(II)–hexadecafluorophthalocyanine (15.7 mg, 18.3 μmol, 2 mol%), methanesulfonic acid (34.8 mg, 362 μmol) and 1-(diphenylamino)naphthalene (**3**) (265 mg, 897 μmol). A 100 mL splash head was attached on the flask to prevent evaporation of the solvent, while ensuring sufficient gas exchange. The resulting solution was stirred at room temperature for 24 h under air. Then, 10 mL of a saturated aqueous solution of sodium hydrogen carbonate was added. The aqueous layer was extracted three times with dichloromethane. The combined organic layers were dried (magnesium sulfate). The solvent was evaporated and the residue was purified by automated flash chromatography (silica gel, isohexane/dichloromethane, 20% to 50% in 1.5 h) to provide *N*,*N*,*N*′,*N*′-tetraphenylnaphthidine (**4**) (128 mg, 217 µmol, 48%) as a colorless solid (less polar fraction) and naphthidine **5** (29.5 mg, 33.4 µmol, 11%) as a colorless solid (more polar fraction). Crystallization of compound **4** from isohexane afforded colorless crystals which were suitable for X-ray analysis.

*N^4^,N^4^,N^4^’,N^4^’-Tetraphenyl-[1,1’-binaphthalene]-4,4’-diamine (N*,*N*,*N*′,*N*′-*Tetraphenylnaphthidine)* (**4**): M.p. 249–250 °C; UV (MeOH) *λ* = 217, 291, and 361 nm; fluorescence (MeOH) *λ_ex_* = 291 and *λ_em_* = 446 nm; IR (ATR) *ν* = 3058, 3034, 1931, 1740, 1582, 1488, 1458, 1420, 1397, 1376, 1266, 1153, 1075, 1053, 1025, 920, 836, 746, 691, and 624 cm^−1^; ^1^H-NMR (600 MHz, CDCl_3_) *δ* = 6.98 (t, *J* = 7.3 Hz, 4H), 7.14 (dd, *J* = 8.7, 1.1 Hz, 8H), 7.23–7.28 (m, 8H), 7.31 (ddd, *J* = 8.4, 6.9, 1.3 Hz, 2H), 7.36 (ddd, *J* = 8.4, 6.9, 1.3 Hz, 2H), 7.44 (d, *J* = 7.3 Hz, 2H), 7.50 (d, *J* = 7.9 Hz, 2H), 7.52 (d, *J* = 7.3 Hz, 2H), and 8.08 (d, *J* = 8.1 Hz, 2H); ^13^C-NMR and DEPT at θ = 135° (150 MHz, CDCl_3_) *δ* = 121.93 (4 CH), 122.17 (8 CH), 124.67 (2 CH), 126.35 (2 CH), 126.43 (2 CH), 126.81 (2 CH), 127.32 (2 CH), 128.71 (2 CH), 129.32 (8 CH), 131.40 (2 C), 134.72 (2 C), 136.76 (2 C), 143.55 (2 C), and 148.70 (4 C); MS (ESI, +10 V) *m/z* = 589.3 [M + H]^+^; elemental analysis calcd. for C_44_H_32_N_2_, C 89.76, H 5.48, and N 4.76; found, C 89.61, H 5.63, and N 4.73.

Crystallographic data for *N*,*N*,*N*’,*N*’-tetraphenylnaphthidine (**4**): C_44_H_32_N_2_, crystal size 0.100 × 0.102 × 0.522 mm^3^, *M* = 588.71 g mol^−1^, orthorhombic, space group *P*2_1_2_1_2_1_, *a* = 7.6835(4), *b* = 12.5020(6), *c* = 33.1785(18) Å, *V* = 3187(3) Å^3^, *Z* = 4, *ρ_calcd._* = 1.227 g cm^−3^, *µ* = 0.071 mm^−1^, *T* = 150(2) K, *λ* = 0.71073 Å, *θ* range 2.04–28.34°, 50987 reflections collected, 7939 independent (*R*_int_ = 0.0473), and 415 parameters. The structure was solved by direct methods and refined by full-matrix least-squares on *F*^2^; *R*_1_ = 0.0391 and *wR*_2_ = 0.0890 [*I* > 2*σ*(*I*)]; maximal residual electron density 0.172 e Å^−3^. The absolute structure was not determined. CCDC 1983355.

*N^4^-(4-(4-(Diphenylamino)naphthalen-1-yl)phenyl)-N^4^,N^4^’,N^4^’-triphenyl-[1,1’-binaphthalene]-4,4’-diamine* (**5**): M.p. 272–275 °C; UV (MeOH) *λ* = 215, 291, and 367 nm; fluorescence (MeOH) *λ_ex_* = 291 and *λ_em_* = 453 nm; IR (ATR) *ν* = 3061, 3035, 2952, 2920, 2851, 1727, 1585, 1488, 1457, 1420, 1377, 1265, 1174, 1154, 1074, 1026, 952, 920, 827, 748, 692, and 625 cm^−1^; ^1^H-NMR (600 MHz, CDCl_3_) *δ* = 6.94 (t, *J* = 7.3 Hz, 2H), 6.99 (t, *J* = 7.3 Hz, 2H), 7.03 (t, *J* = 7.3 Hz, 1H), 7.06–7.11 (m, 4H), 7.13–7.17 (m, 4H), 7.19–7.30 (m, 12H), 7.32 (t, *J* = 8.1 Hz, 3H), 7.34–7.39 (m, 4H), 7.40–7.48 (m, 6H), 7.49–7.61 (m, 5H), 8.03 (d, *J* = 8.3 Hz, 1H), 8.09 (t, *J* = 7.7 Hz, 2H), and 8.18 (d, *J* = 8.7 Hz, 1H); ^13^C-NMR and DEPT at θ = 135° (150 MHz, CDCl_3_) *δ* = 121.40 (2 CH), 121.78 (2 CH), 121.95 (2 CH), 122.00 (4 CH), 122.19 (4 CH), 122.28 (CH), 122.54 (2 CH), 124.66 (CH), 124.70 (CH), 124.71 (CH), 126.19 (CH), 126.31 (CH), 126.39 (CH), 126.45 (CH), 126.46 (CH), 126.57 (CH), 126.82 (CH), 126.93 (CH), 127.03 (CH), 127.05 (CH), 127.31 (CH), 127.41 (CH), 127.55 (CH), 128.74 (CH), 128.79 (CH), 129.23 (4 CH), 129.33 (4 CH), 129.44 (2 CH), 131.07 (2 CH), 131.41 (C), 131.51 (C), 131.67 (C), 133.51 (C), 133.72 (C), 134.72 (C), 134.80 (C), 136.72 (C), 137.03 (C), 138.64 (C), 142.80 (C), 143.39 (C), 143.61 (C), 147.93 (C), 148.46 (C), 148.61 (2 C), and 148.70 (2 C); MS (ESI, +10 V) *m/z* = 882.5 [M + H]^+^; elemental analysis calcd. for C_66_H_47_N_3_, C 89.87, H 5.37, and N 4.76; found, C 89.56, H 5.31, and N 4.79.

## 4. Conclusions

In conclusion, we have developed a two-step synthesis of *N*,*N*,*N*’,*N*’-tetraphenylnaphthidine (**4**). Starting from diphenylamine (**1**), Buchwald–Hartwig coupling with 1-bromonaphthalene (**2**) and subsequent iron-catalyzed oxidative homocoupling of the resulting 1-(diphenylamino)naphthalene (**3**) provides compound **4** as the major product and as a minor product compound **5**, resulting from an additional oxidative C–C coupling. Thus, we could demonstrate that our method of iron-catalyzed oxidative C–C coupling with air as the final oxidant can be applied to the regioselective homocoupling of triarylamines. Compounds **4** and **5** exhibit a strong blue-light fluorescence with quantum yields of up to 68% and fluorescence lifetimes of 4.2 and 3.6 ns, for compounds **4** and **5**, respectively. Further structural changes of the *N*,*N*,*N*’,*N*’-tetraarylnaphthidines by extension of the π-system or modification of the substitution pattern could lead to improved photophysical properties and fluorophores for various potential applications.

## Figures and Tables

**Table 1:** UV absorption and fluorescence emission data of the compounds **3**, **4**, and **5** in methanol.

Compound	λ_abs_ (nm) ^a^	ε (M^−1^ cm^−1^) ^b^	λ_em_ (nm) ^c^	Δλ (nm) ^d^
aminonaphthalene **3**	290	15,700	439	149
naphthidine **4**	291	11,400	446	155
naphthidine **5**	291	6000	453	162

^a^ UV absorption maximum; ^b^ molar extinction coefficient; ^c^ fluorescence emission maximum (excitation at λ_ex_ = 291 nm); ^d^ Stokes shift, Δλ = λ_em_ − λ_abs_.

**Table 2:** Photophysical data of the naphthidines **4** and **5**.

Compound	λ_abs_ (nm) ^a^	ε (M^−1^ cm^−1^) ^b^	λ_em_ (nm) ^c^	Φ_fl_^d,e^	т (ns) ^f,e^	CIE-coord. (x; y) ^g,e^
naphthidine **4**	294	32,000	449	0.68	4.2	(0.153; 0.054)
naphthidine **5**	294	25,600	453	0.65	3.6	(0.152; 0.062)

^a^ UV absorption maximum in CH_2_Cl_2_; ^b^ molar extinction coefficient in CH_2_Cl_2_; ^c^ fluorescence emission maximum in CH_2_Cl_2_ (λ_ex_ = 294 nm); ^d^ fluorescence quantum yield (λ_ex_ = 310 nm); ^e^ in a 2% PMMA film; ^f^ fluorescence lifetime (λ_ex_ = 360 nm); ^g^ Commission internationale de l’éclairage (CIE) (λ_ex_ = 310 nm).

**Table 3:** UV absorption and fluorescence emission data of the naphthidine **4** in different solvents.

	Isohexane	CH_2_Cl_2_	Ethyl Acetate	THF	Methanol
λ_abs_ (nm) ^a^	291	294	291	292	291
λ_em_ (nm) ^b^	428	449	439	445	446

^a^ UV absorption maximum and ^b^ fluorescence emission maximum (for each solvent λ_ex_ = λ_abs_).

## Supplementary Materials

The following data are available online. Copies of the ^1^H-NMR, ^13^C-NMR, DEPT (θ = 135°), UV, and fluorescence spectra of the compounds **3**, **4**, and **5**; copies of the 2D-NMR spectra (COSY, HSQC, HMBC, NOESY, ^1^H/^15^N-HMBC, JRES) of the compounds **4** and **5**.
