# Supplementary material for: Iron-Catalyzed Synthesis, Structure, and Photophysical Properties of Tetraarylnaphthidines
Source: Molecules. 2020 Apr 1;25(7):1608. doi: 10.3390/molecules25071608 (PMC7181034; doi:10.3390/molecules25071608)
Supplement: Supplementary file 1 [file molecules-25-01608-s001.pdf]

Supplementary Material

Iron-catalyzed Synthesis, Structure, and Photophysical Properties of Tetraarylnaphthidines

Alexander Purtsas [1], Sergej Stipurin [1], Olga Kataeva [2] and Hans-Joachim Knölker [1,*]

[1] Faculty of Chemistry, Technische Universität Dresden, Bergstraße 66, 01069 Dresden, Germany; alexander.purtsas@chemie.tu-dresden.de (A.P.); sergej.stipurin@chemie.tu-dresden.de (S.S.)

[2] A. E. Arbuzov Institute of Organic and Physical Chemistry, FRC Kazan Scientific Center, Russian Academy of Sciences, Arbuzov Str. 8, Kazan 420088, Russia; olga-kataeva@yandex.ru

[*] Correspondence: hans-joachim.knoelker@tu-dresden.de; Fax +49-351-463-37030

Contents

	Page
^{1}H NMR spectrum of compound **3**	3
DEPT (θ = 135°) and ^{13}C NMR spectrum of compound **3**	4
UV and Fluorescence spectrum of compound **3** in MeOH	5
^{1}H NMR spectrum of compound **4**	6
DEPT (θ = 135°) and ^{13}C NMR spectrum of compound **4**	7
COSY spectrum of compound **4**	8
HSQC spectrum of compound **4**	9
HMBC spectrum of compound **4**	10
NOESY spectrum of compound **4**	11
^{1}H/^{15}N HMBC spectrum of compound **4**	12
Table S1 (^{1}H, ^{13}C NMR data, and HMBC correlations of compound **4**)	13
UV and Fluorescence spectrum of compound **4** in isohexane	14
UV and Fluorescence spectrum of compound **4** in CH_2Cl_2	15
UV and Fluorescence spectrum of compound **4** in EtOAc	16
UV and Fluorescence spectrum of compound **4** in THF	17
UV and Fluorescence spectrum of compound **4** in MeOH	18
^{1}H NMR spectrum of compound **5**	19
DEPT (θ = 135°) and ^{13}C NMR spectrum of compound **5**	20
DEPT (θ = 135°) and ^{13}C NMR spectrum of compound **5** (enlarged section)	21

COSY spectrum of compound **5** 22
COSY spectrum of compound **5** (enlarged section) 23
COSY spectrum of compound **5** (enlarged section) 24
COSY spectrum of compound **5** (enlarged section) 25
HSQC spectrum of compound **5** 26
HSQC spectrum of compound **5** (enlarged section) 27
HSQC spectrum of compound **5** (enlarged section) 28
HSQC spectrum of compound **5** (enlarged section) 29
HSQC spectrum of compound **5** (enlarged section) 30
HMBC spectrum of compound **5** 31
HMBC spectrum of compound **5** (enlarged section) 32
NOESY spectrum of compound **5** 33
NOESY spectrum of compound **5** (enlarged section) 34
NOESY spectrum of compound **5** (enlarged section) 35
$^{1}H/^{15}N$ HMBC spectrum of compound **5** 36
JRES spectrum of compound **5** 37
JRES spectrum of compound **5** (enlarged section) 38
Table S2 (^{13}C NMR data of compound **5**) 39
UV and Fluorescence spectrum of compound **5** in CH_2Cl_2 40
UV and Fluorescence spectrum of compound **5** in MeOH 41

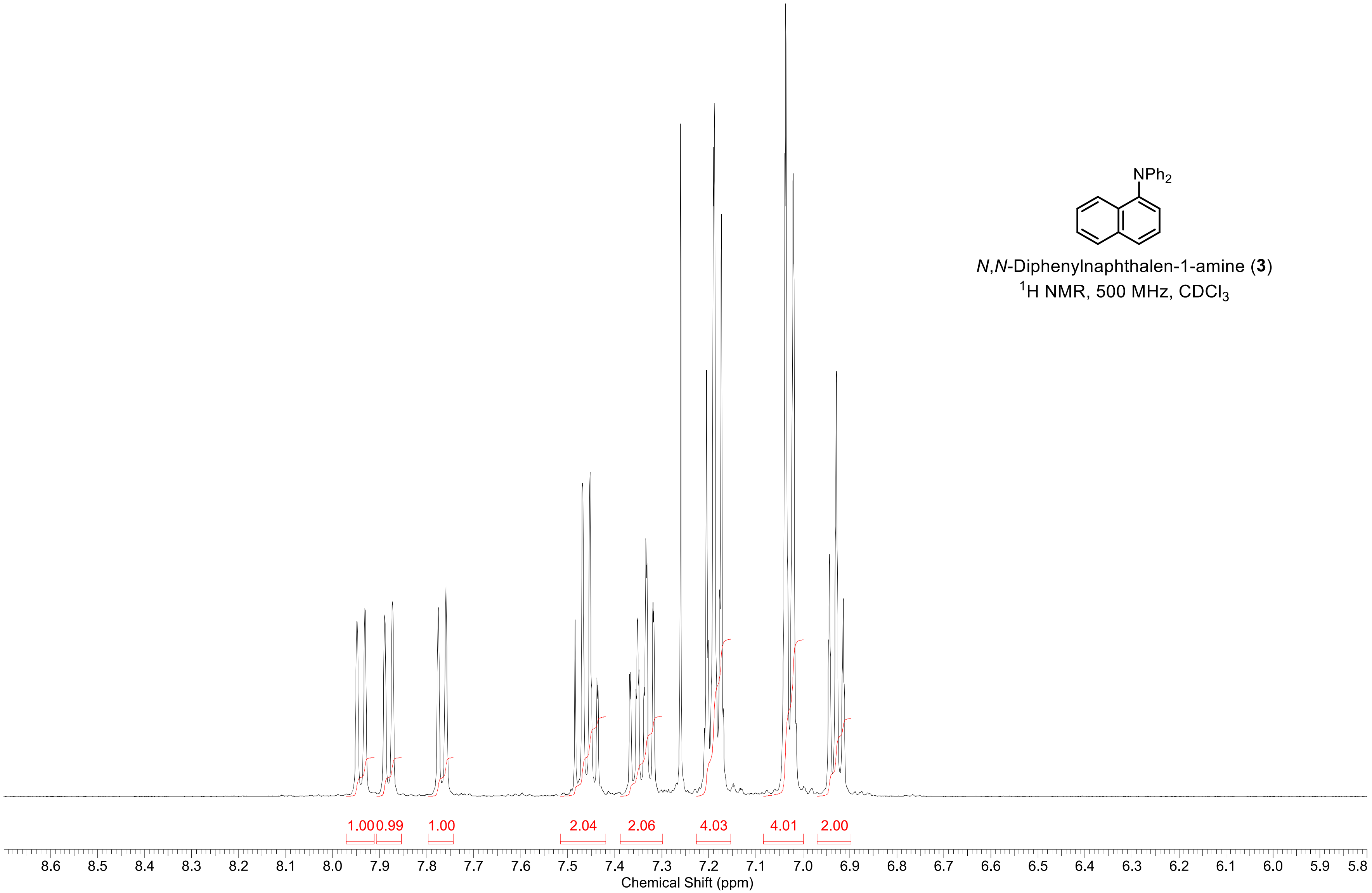

N,*N*-Diphenylnaphthalen-1-amine (**3**)

^{1}H NMR, 500 MHz, $CDCl_3$

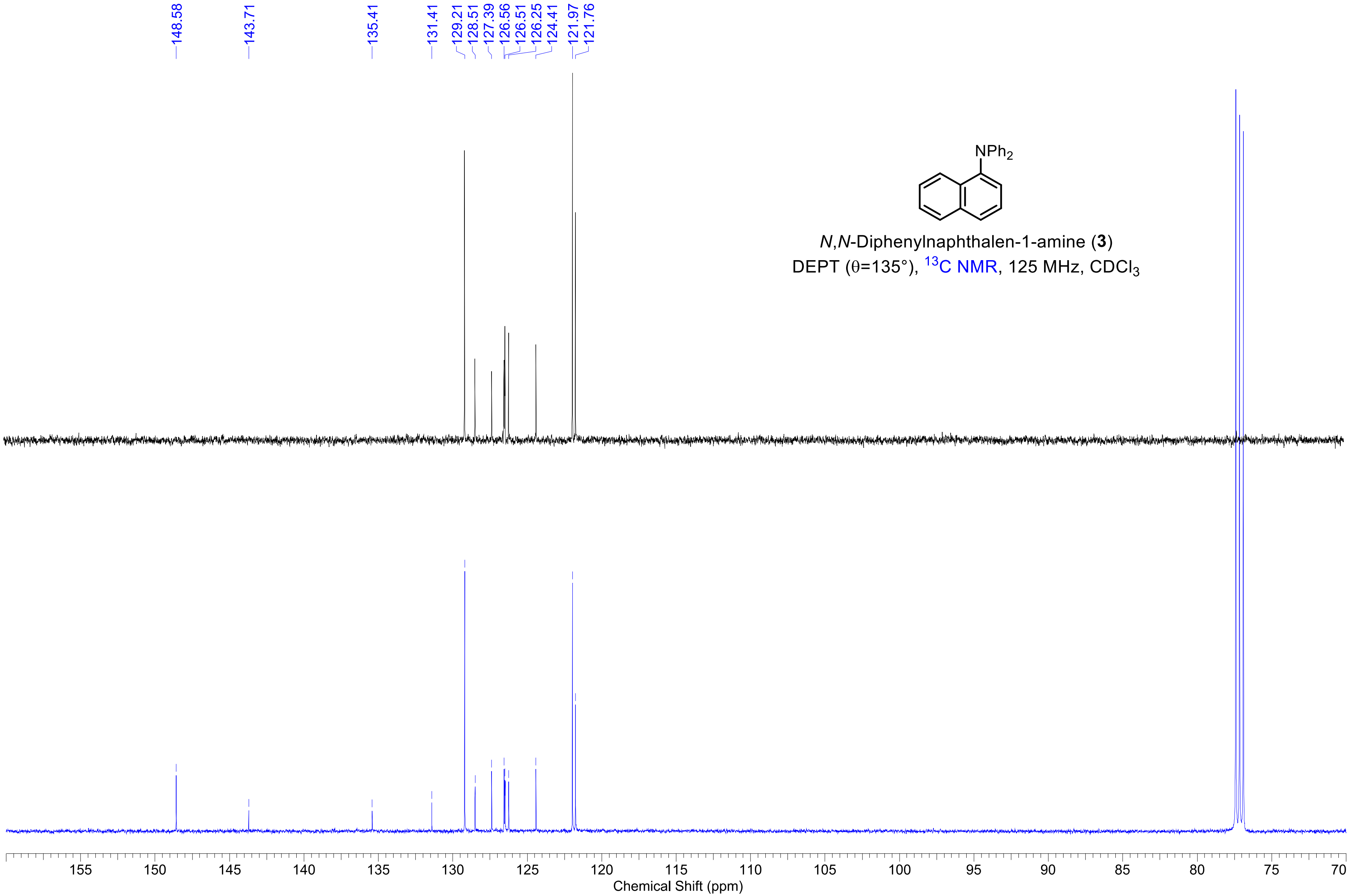
148.58
143.71
135.41
131.41
129.21
128.51
127.39
126.56
126.51
126.25
124.41
121.97
121.76
NPh2
N,N-Diphenylnaphthalen-1-amine (3)
DEPT (θ=135°), 13C NMR, 125 MHz, CDCl3
155
150
145
140
135
130
125
120
115
110
105
100
95
90
85
80
75
70
Chemical Shift (ppm)

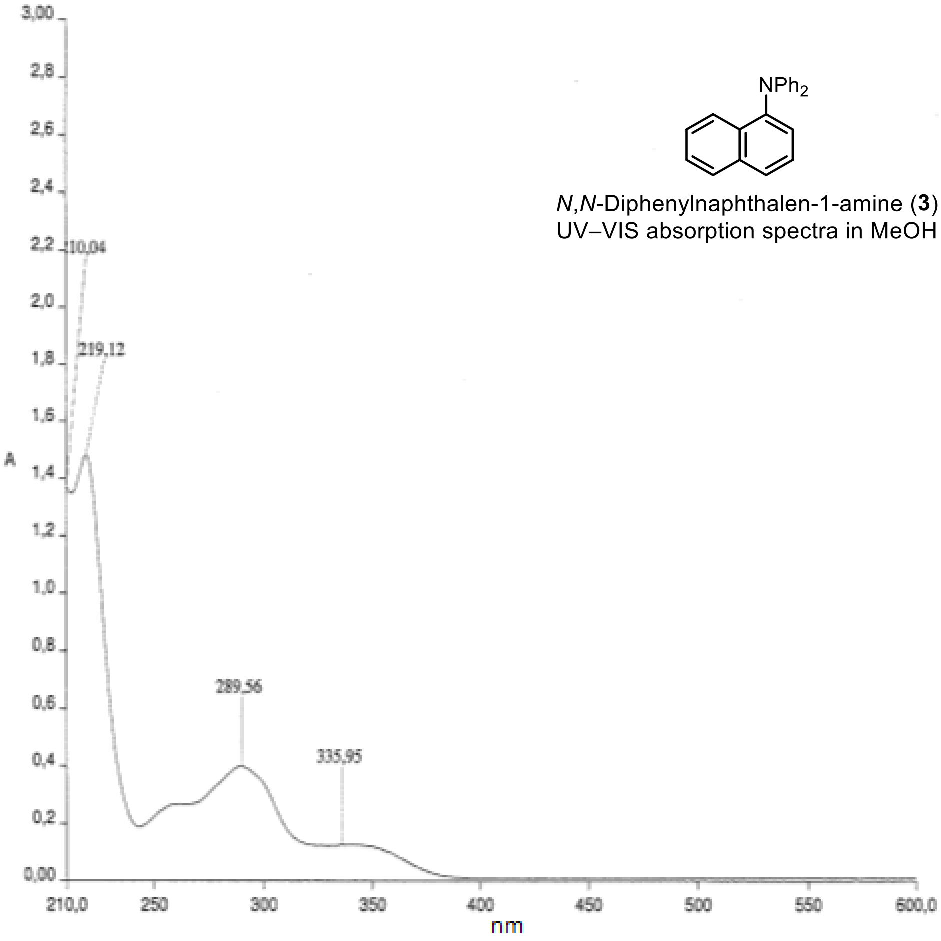

N,*N*-Diphenylnaphthalen-1-amine (**3**)
UV–VIS absorption spectra in MeOH

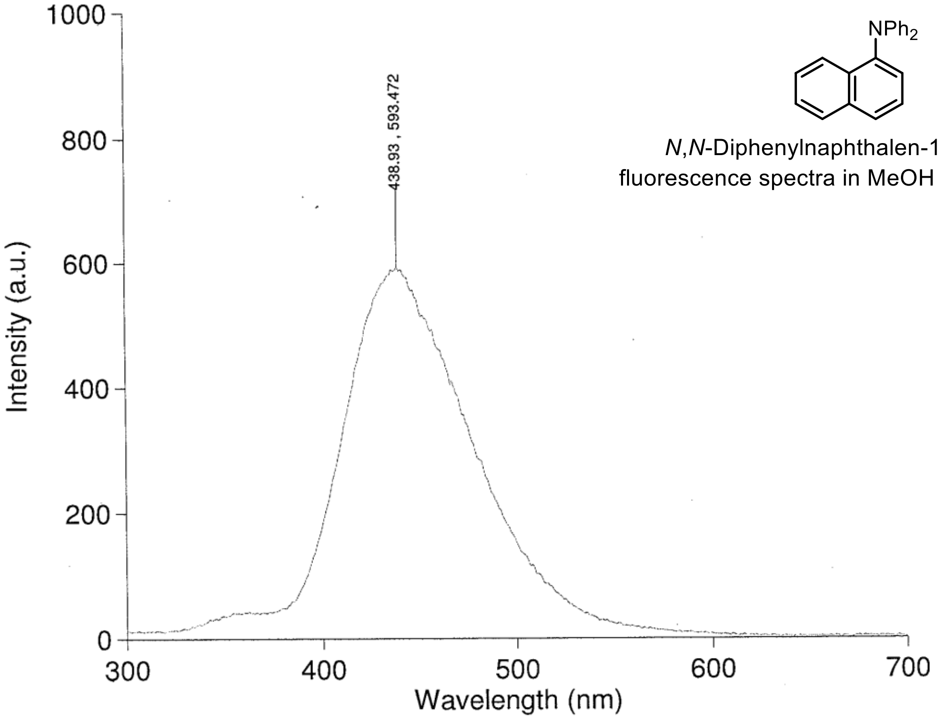

N,*N*-Diphenylnaphthalen-1-amine (**3**)
fluorescence spectra in MeOH (λ_{ex} = 291 nm)

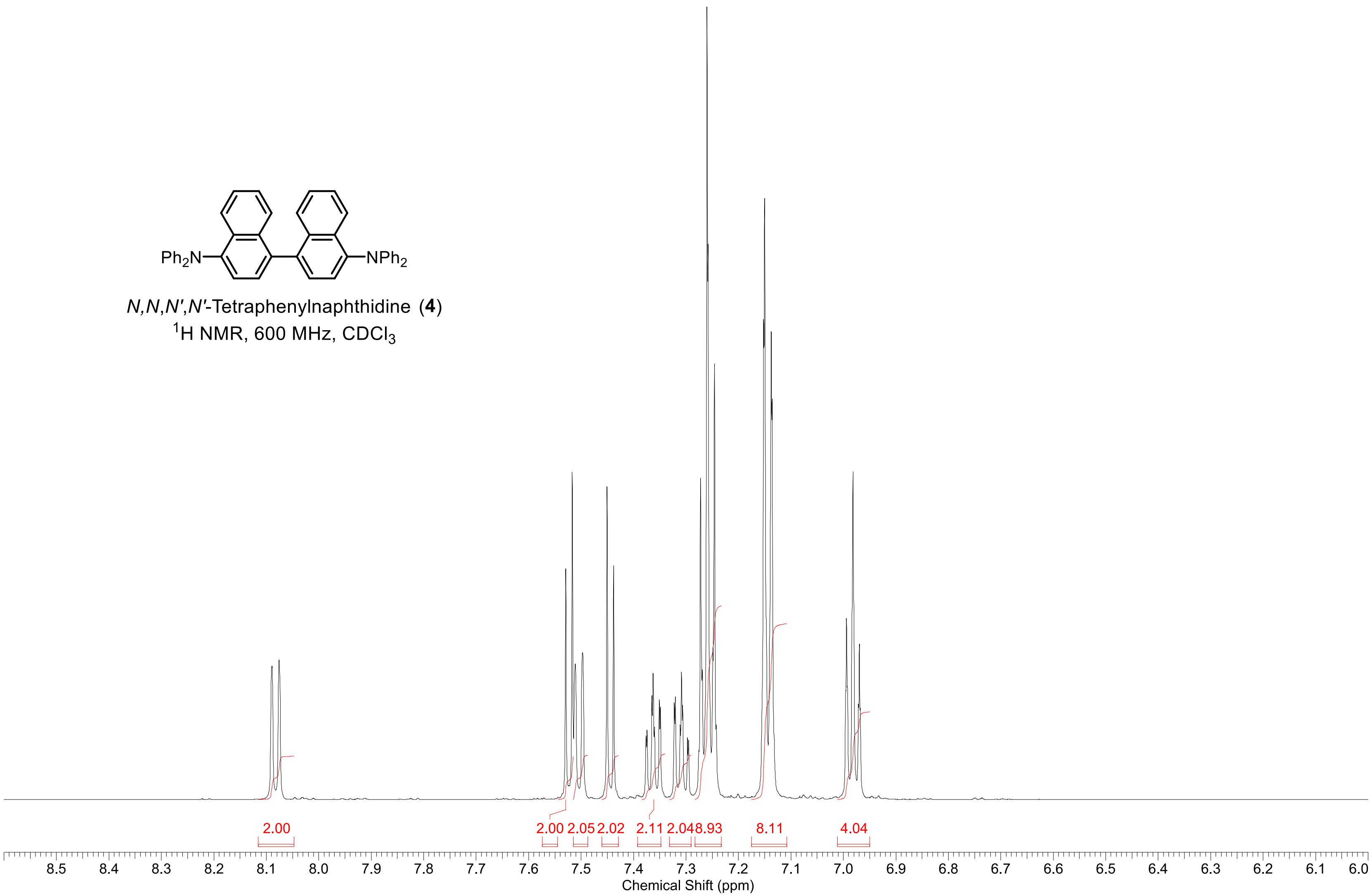

N,N,N′,N′-Tetraphenylnaphthidine (**4**)
^{1}H NMR, 600 MHz, $CDCl_3$

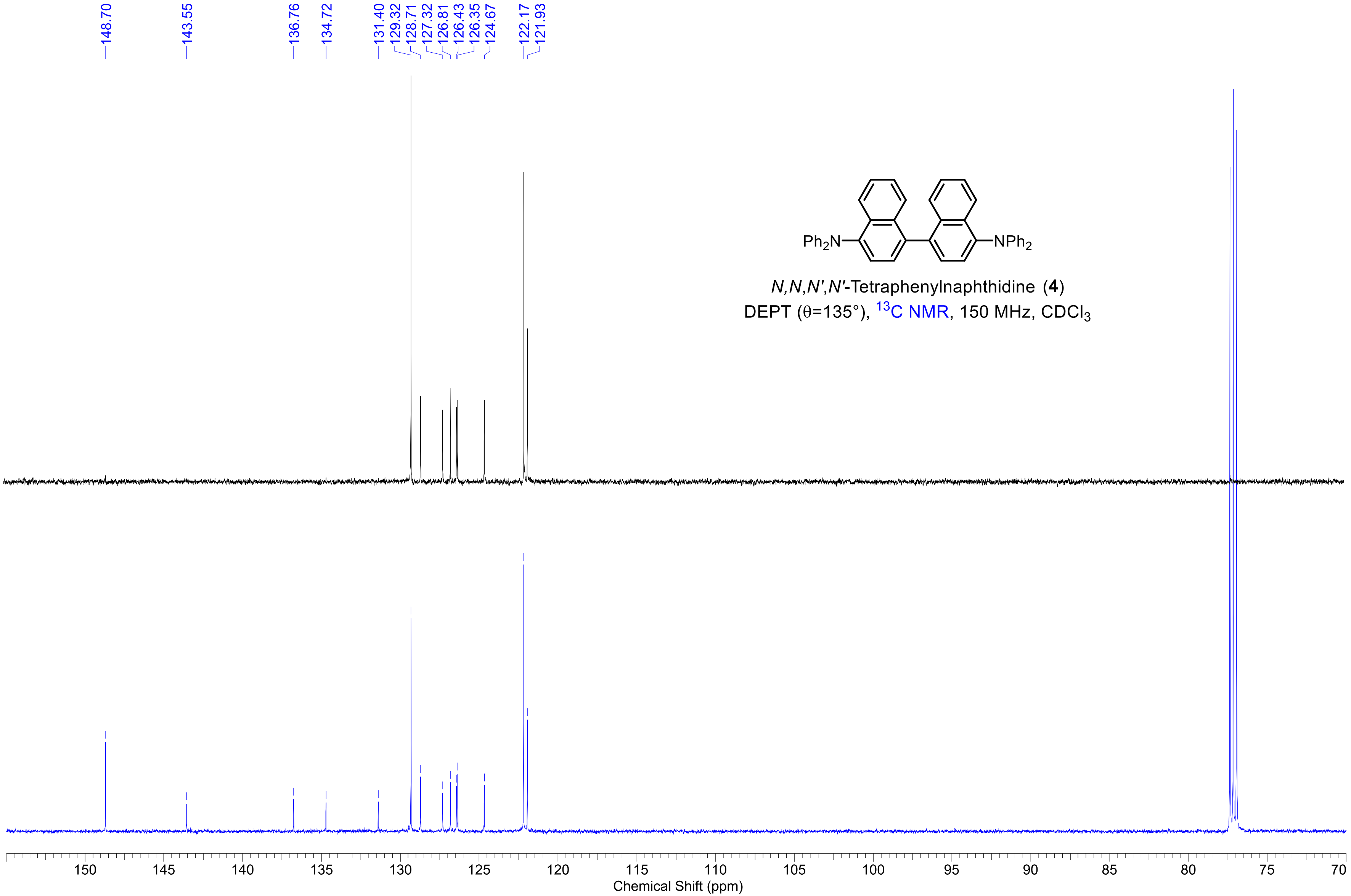

N,N,N′,N′-Tetraphenylnaphthidine (**4**)

DEPT (θ=135°), ^{13}C NMR, 150 MHz, $CDCl_3$

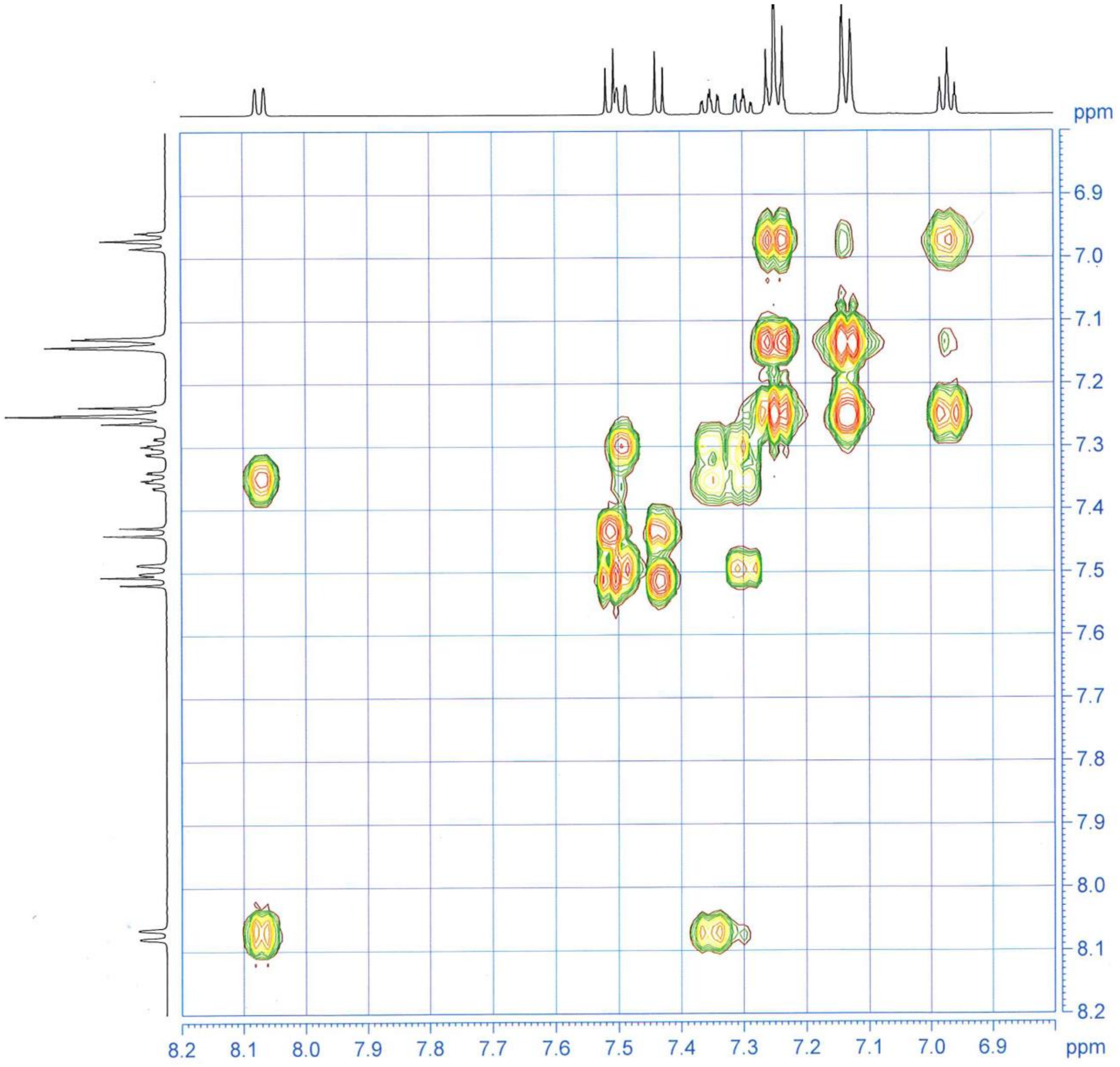

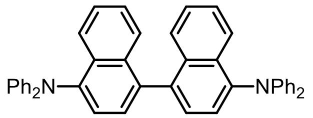

N,N,N′,N′-Tetraphenylnaphthidine (**4**)
COSY, $CDCl_3$

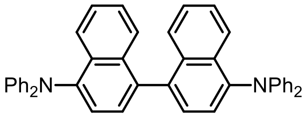

N,*N*,*N′*,*N′*-Tetraphenylnaphthidine (**4**)
HSQC, $CDCl_3$

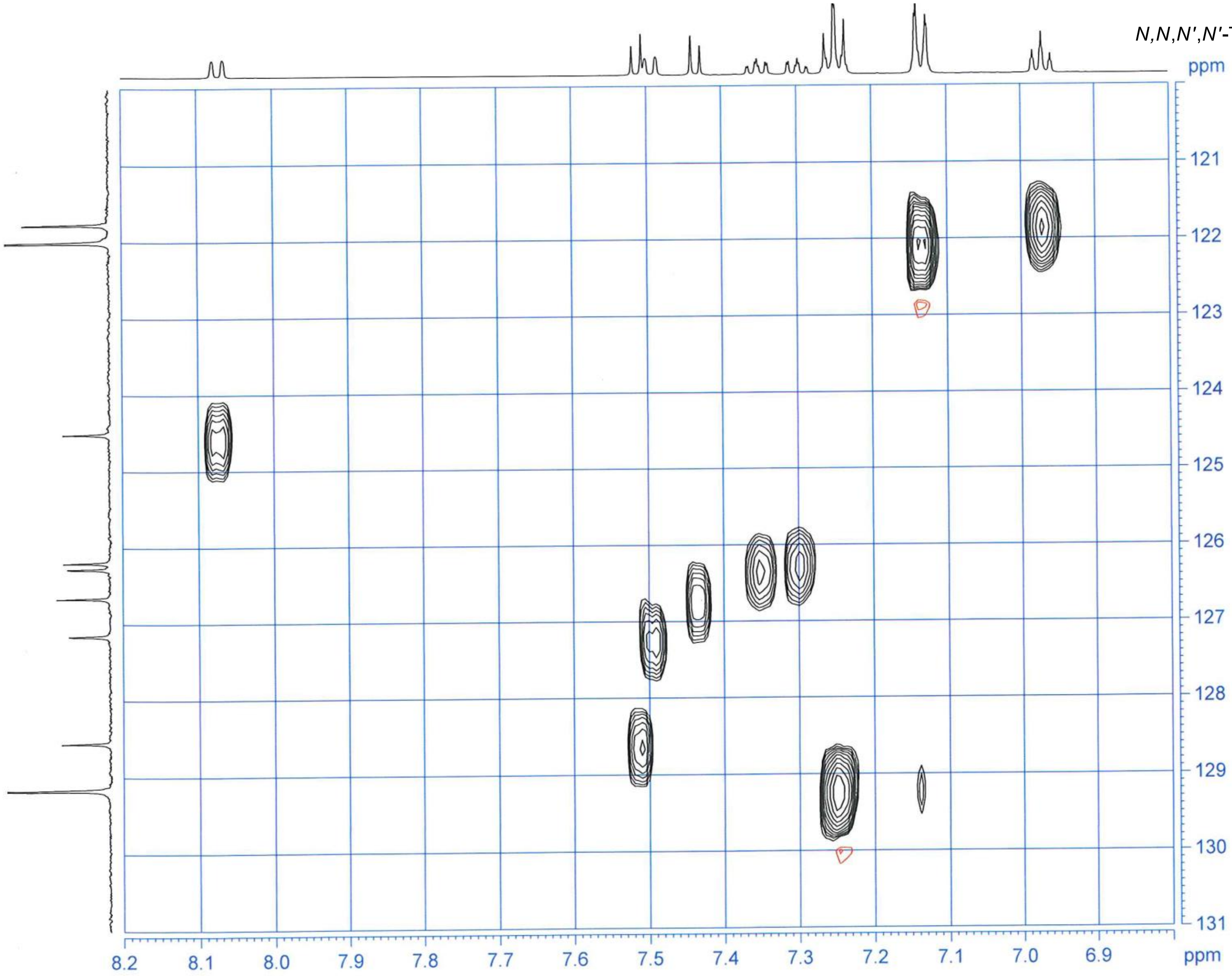

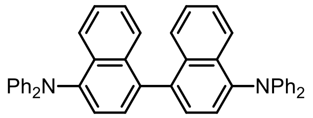

N,*N*,*N′*,*N′*-Tetraphenylnaphthidine (**4**)
HMBC, $CDCl_3$

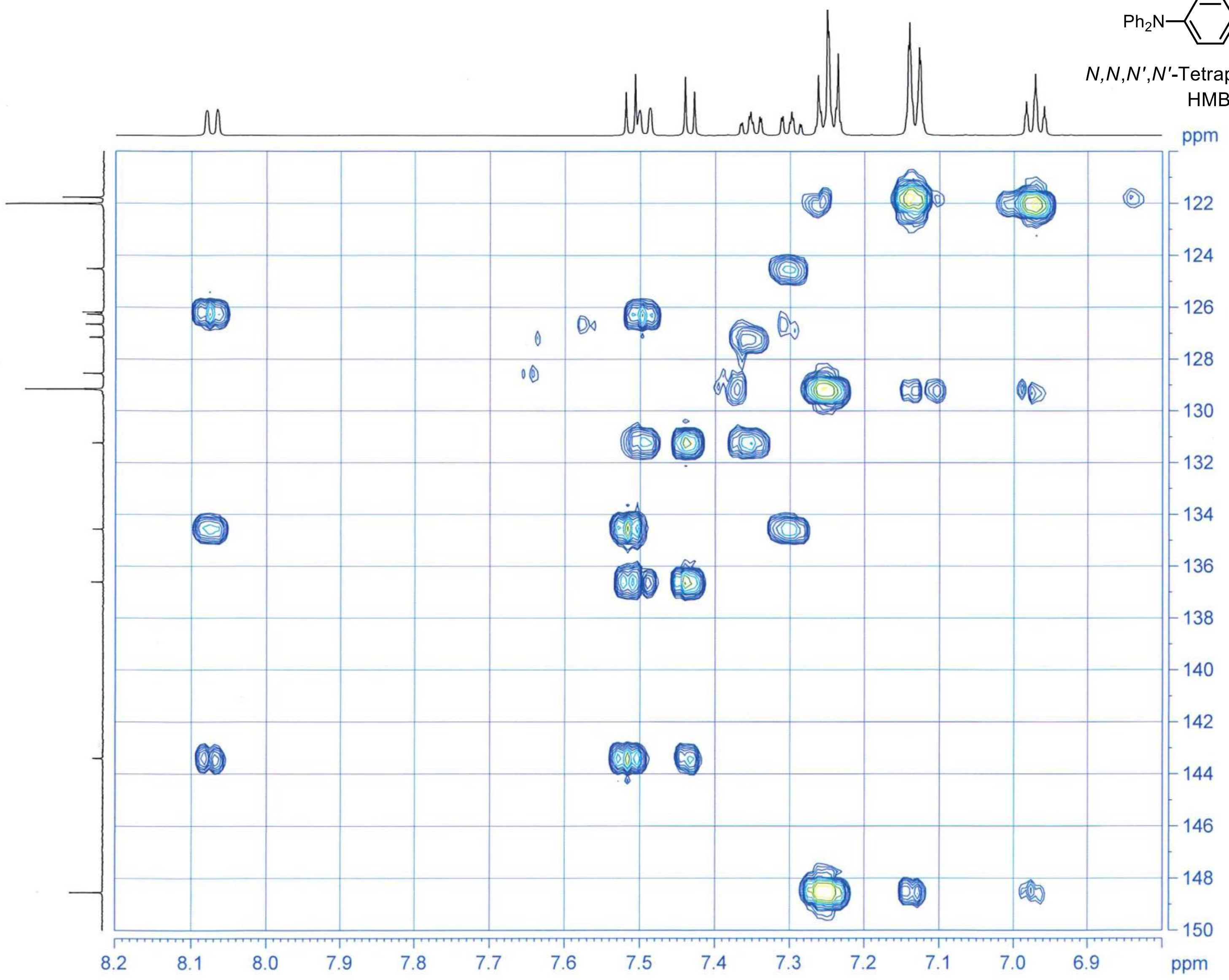

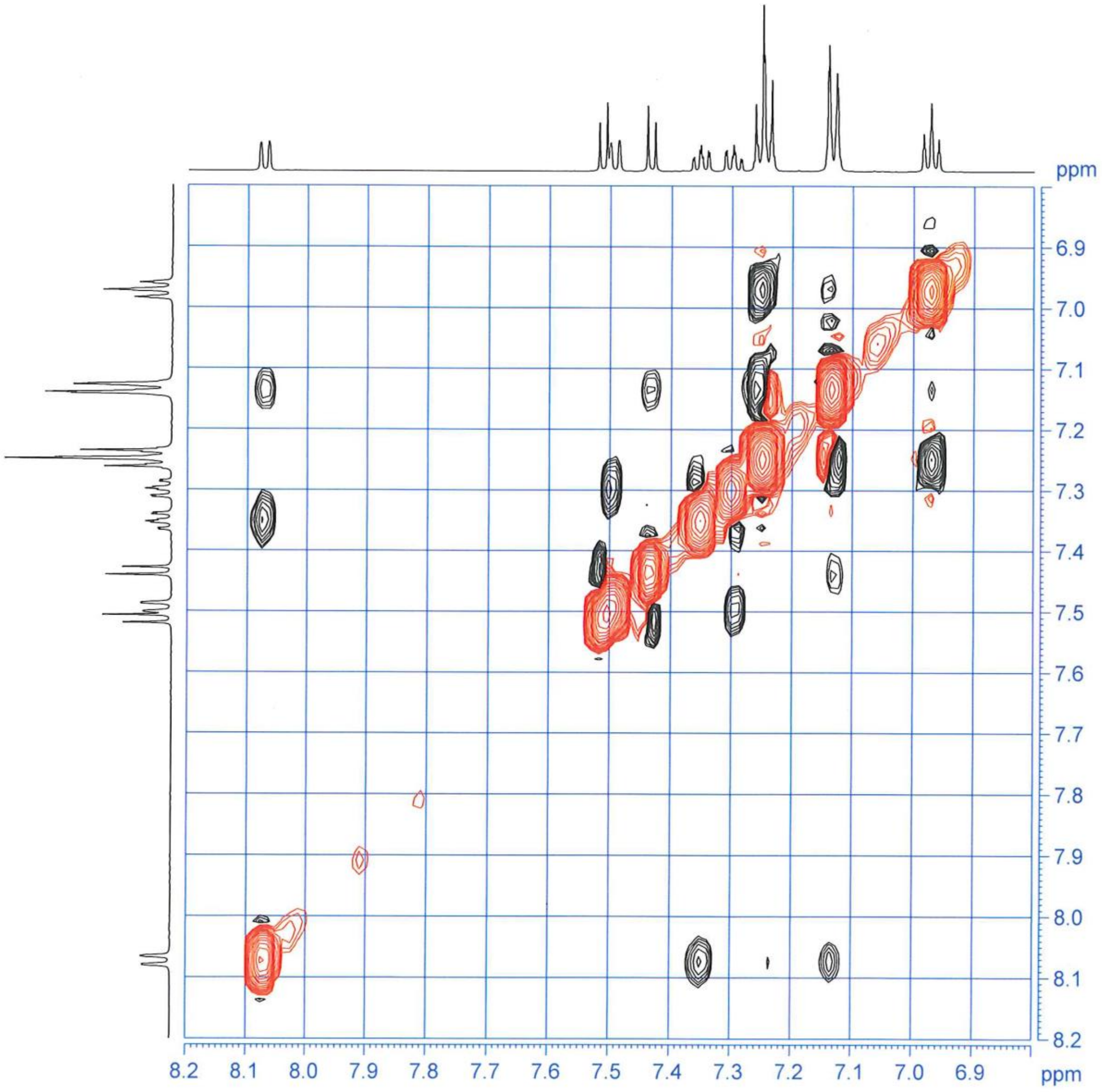

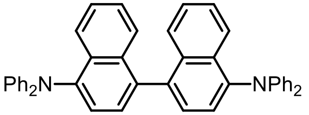

N,*N*,*N′*,*N′*-Tetraphenylnaphthidine (**4**)
NOESY, $CDCl_3$

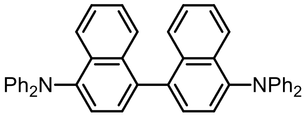

N,*N*,*N′*,*N′*-Tetraphenylnaphthidine (**4**)
^{1}H/ ^{15}N HMBC, 600/ 61 MHz, $CDCl_3$

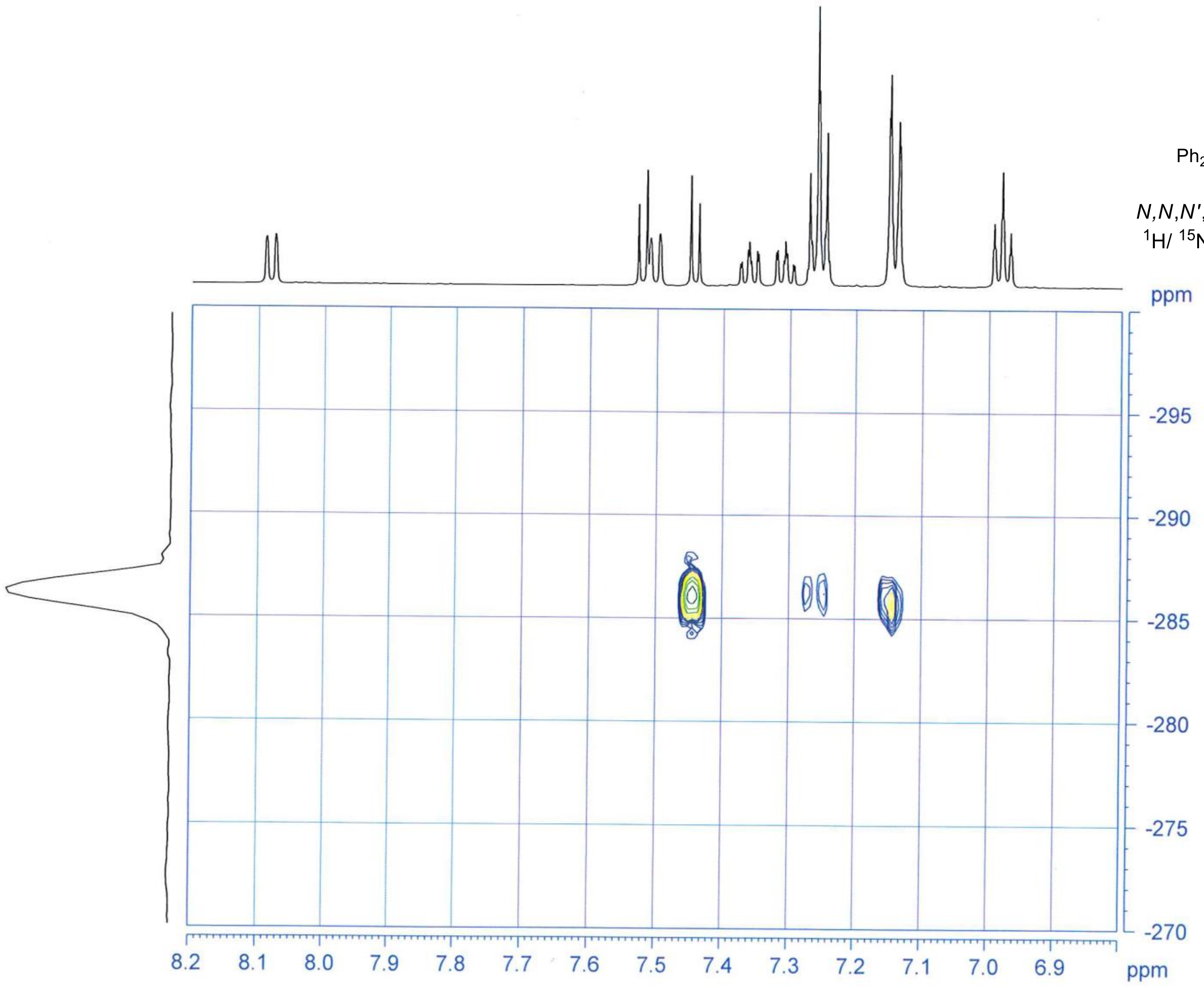

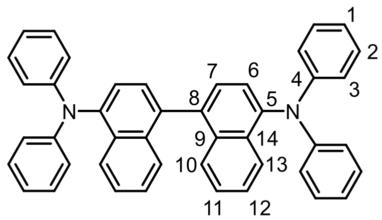

4

Table S1. ^{1}H, ^{13}C NMR data and HMBC correlations of naphthidine **4** in $CDCl_3$.

C	δ_H (*J* in Hz) [a]	δ_C [b]	HMBC [c]
1	6.98 t (7.3)	121.93 CH	3-H
2	7.23–7.28	129.32 CH	2-H
3	7.14 dd (8.7, 1.1)	122.17 CH	1-H, 2-H
4	–	148.70 C	2-H, 3-H
5	–	143.55 C	6-H, 7-H, 13-H
6	7.44 d (7.3)	126.81 CH	–
7	7.52 d (7.3)	128.71 CH	–
8	–	136.76 C	6-H, 7-H, 10-H
9	–	134.72 C	7-H, 11-H, 13-H
10	7.50 d (7.9)	127.32 CH	12-H
11	7.31 ddd (8.4, 6.9, 1.3)	126.35 CH	13-H
12	7.36 ddd (8.4, 6.9, 1.3)	126.43 CH	10-H
13	8.08 d (8.1)	124.67 CH	11-H
14	–	131.40 C	6-H, 10-H, 12-H

[a] ^{1}H NMR data (600 MHz). [b] ^{13}C NMR data (150 MHz) and results of the DEPT experiment (θ = 135°).
[c] HMBC correlations from carbon atoms to protons.

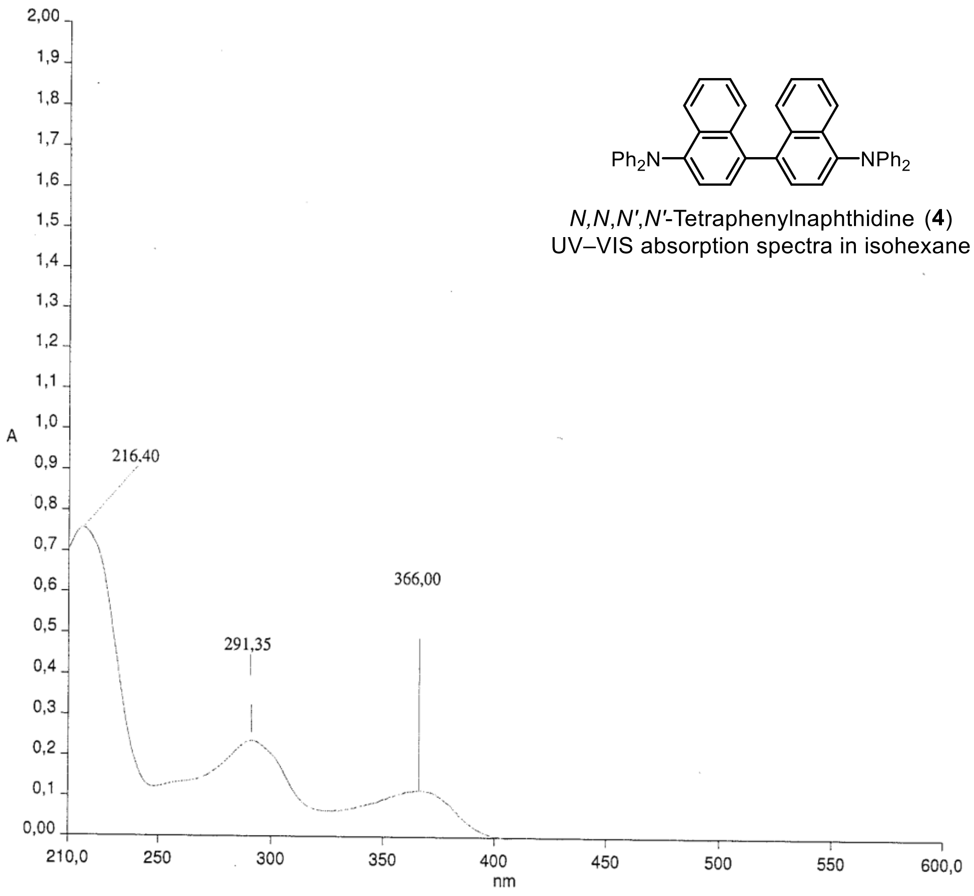

Ph2N
NPh2
N,N,N',N'-Tetraphenylnaphthidine (4)
UV–VIS absorption spectra in isohexane
A
216,40
291,35
366,00
nm

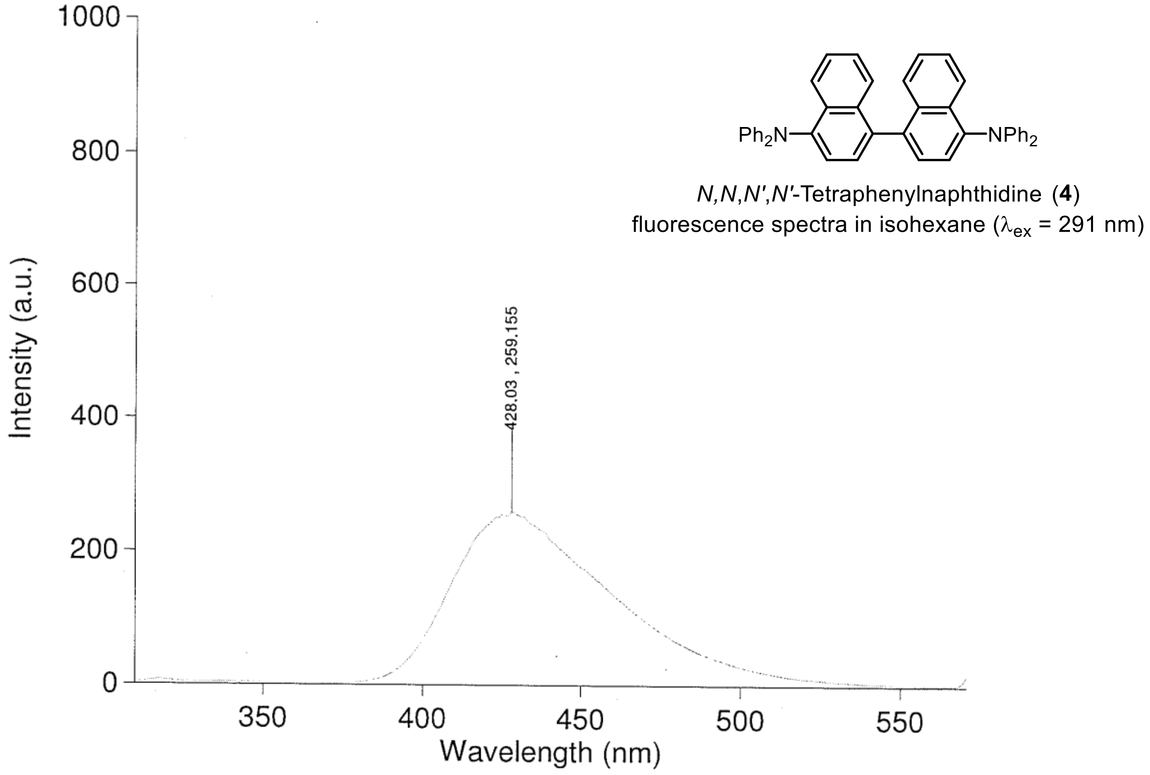

Ph2N
NPh2
N,N,N',N'-Tetraphenylnaphthidine (4)
fluorescence spectra in isohexane (λex = 291 nm)
Intensity (a.u.)
428,03 , 259,155
Wavelength (nm)

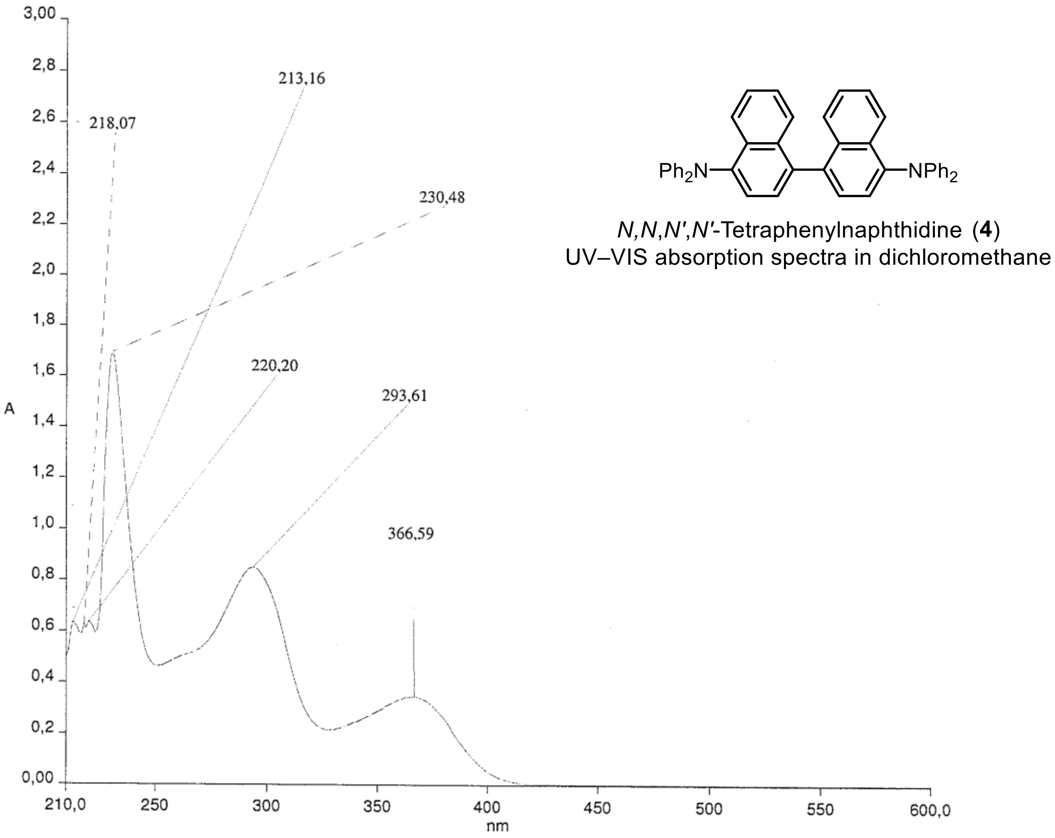

N,N,*N′*,*N′*-Tetraphenylnaphthidine (**4**)
UV–VIS absorption spectra in dichloromethane

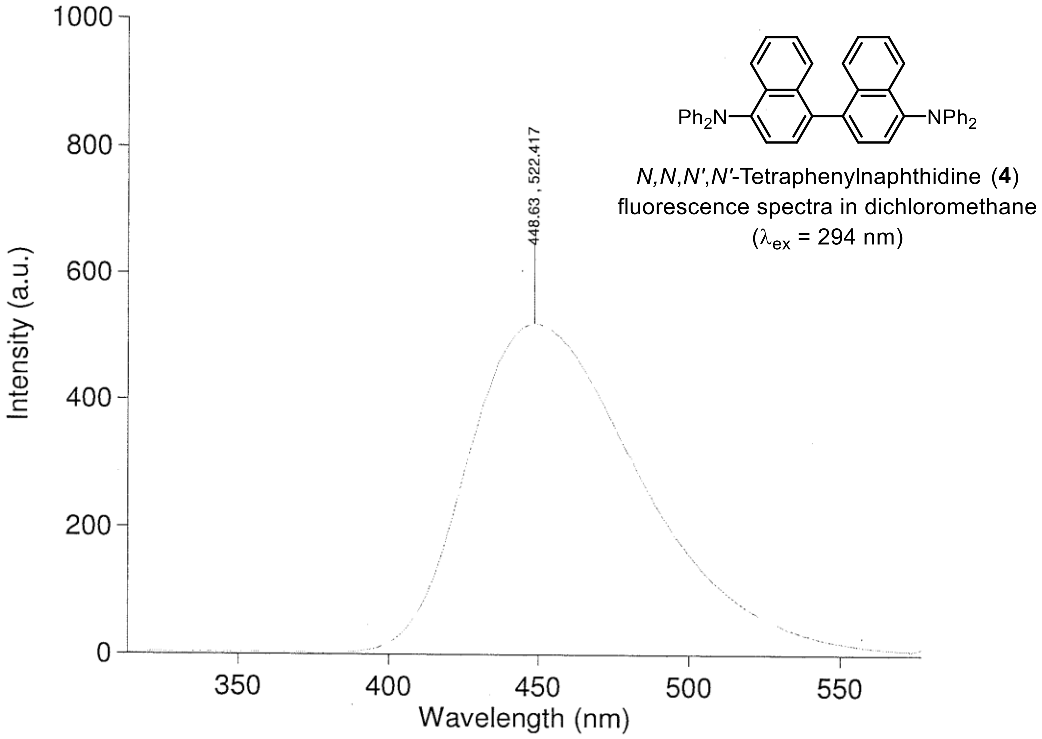

N,N,*N′*,*N′*-Tetraphenylnaphthidine (**4**)
fluorescence spectra in dichloromethane
(λ_{ex} = 294 nm)

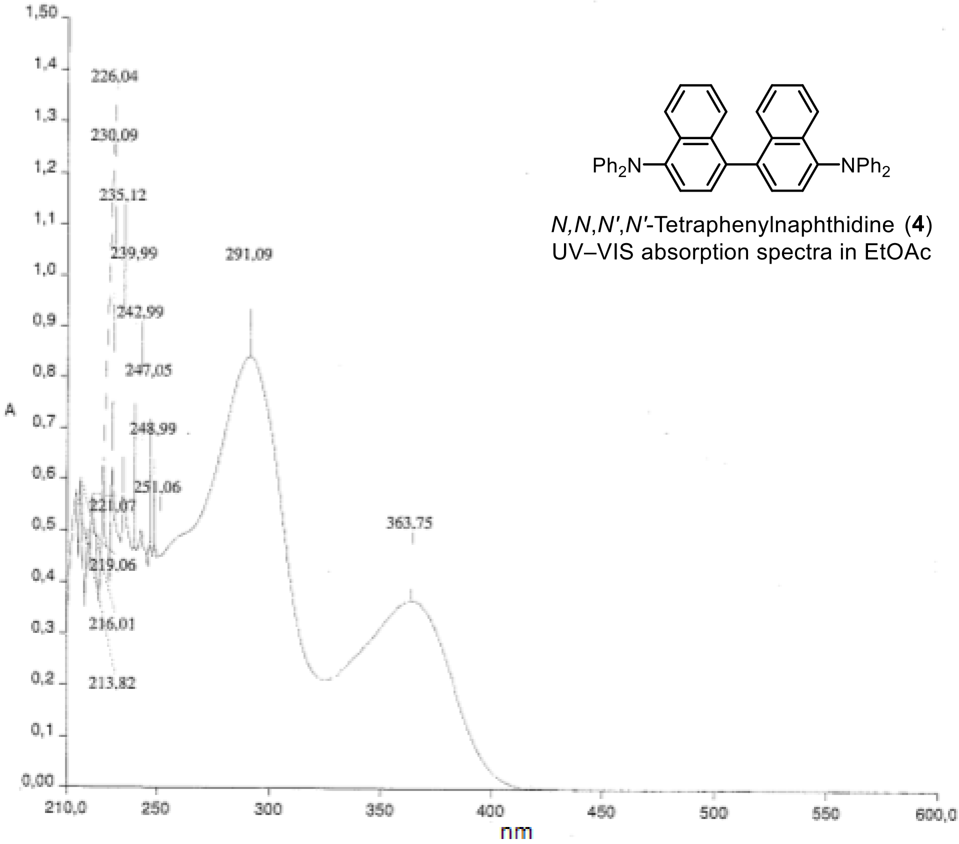

N,*N*,*N′*,*N′*-Tetraphenylnaphthidine (**4**)
UV–VIS absorption spectra in EtOAc

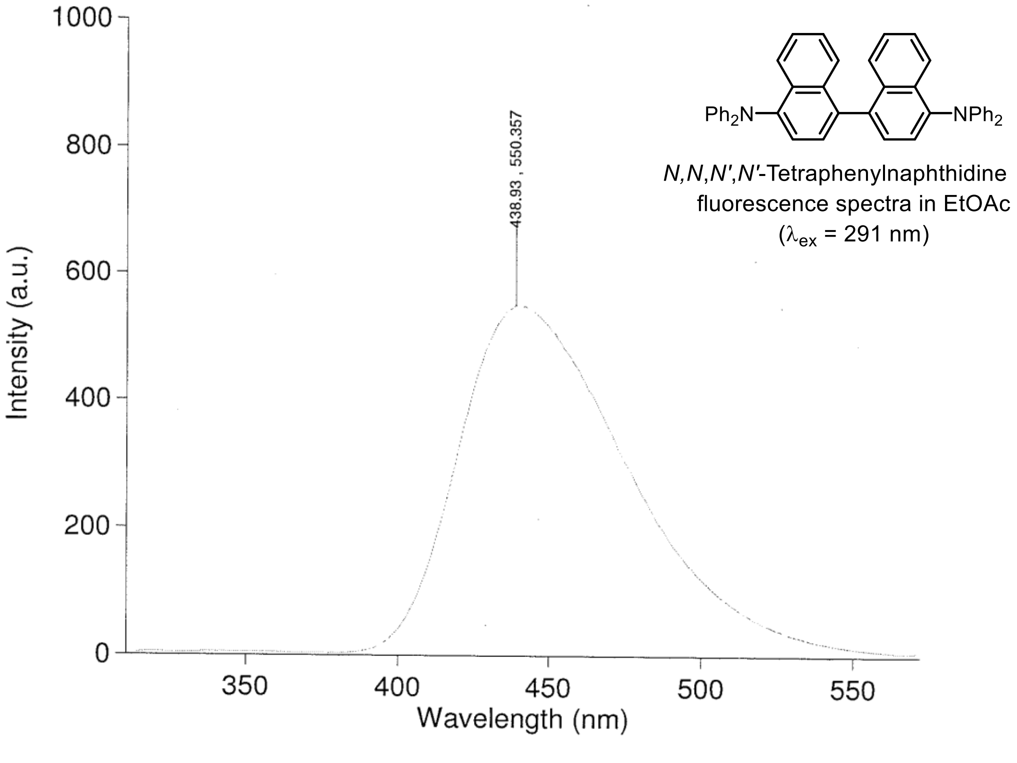

N,*N*,*N′*,*N′*-Tetraphenylnaphthidine (**4**)
fluorescence spectra in EtOAc
(λ_{ex} = 291 nm)

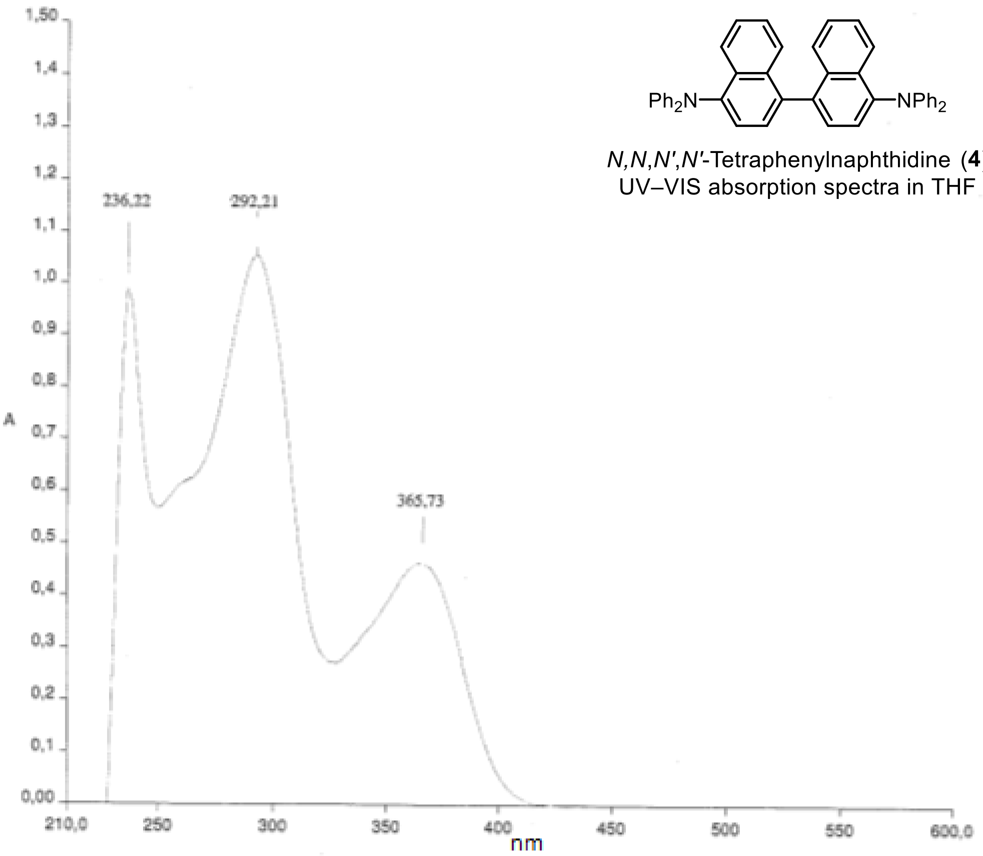

N,*N*,*N'*,*N'*-Tetraphenylnaphthidine (**4**)
UV–VIS absorption spectra in THF

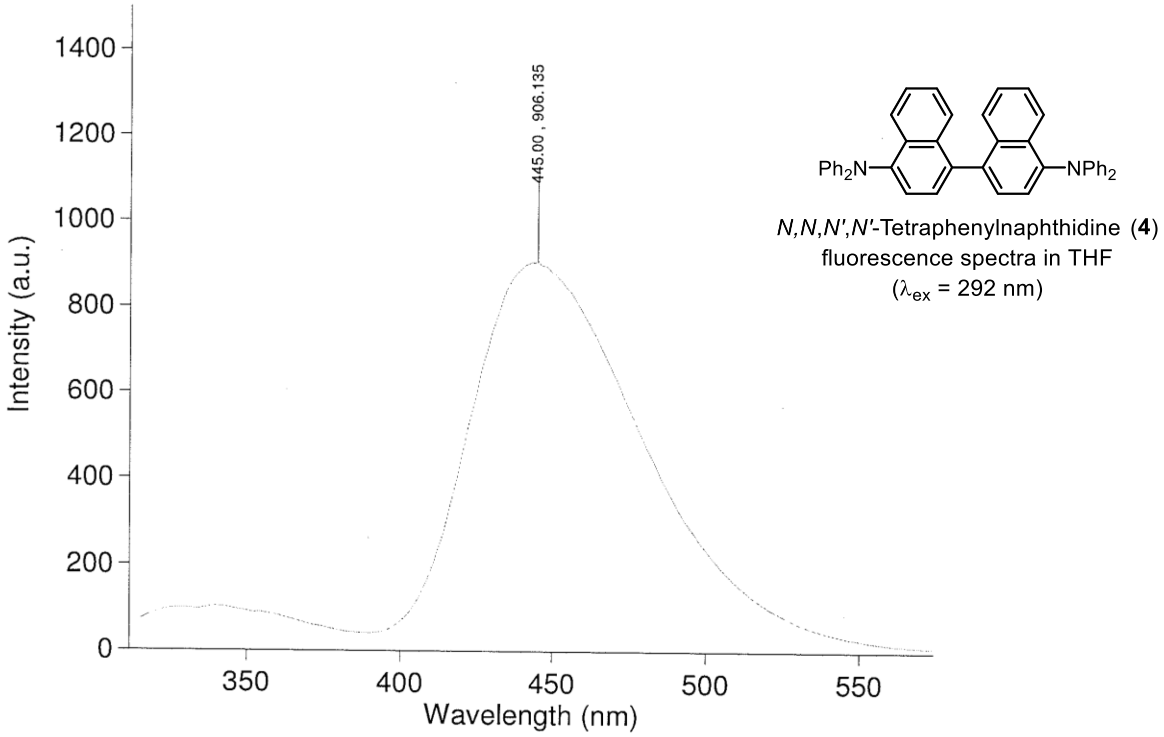

N,*N*,*N'*,*N'*-Tetraphenylnaphthidine (**4**)
fluorescence spectra in THF
(λ_{ex} = 292 nm)

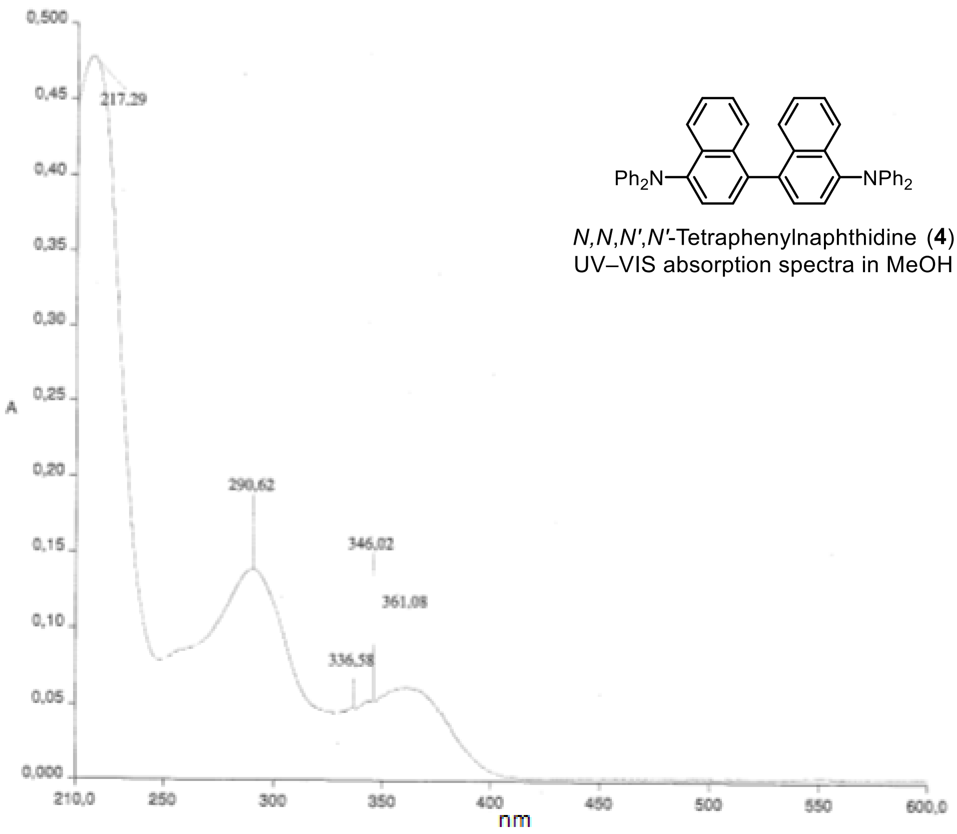

N,N,N′,N′-Tetraphenylnaphthidine (**4**)
UV–VIS absorption spectra in MeOH

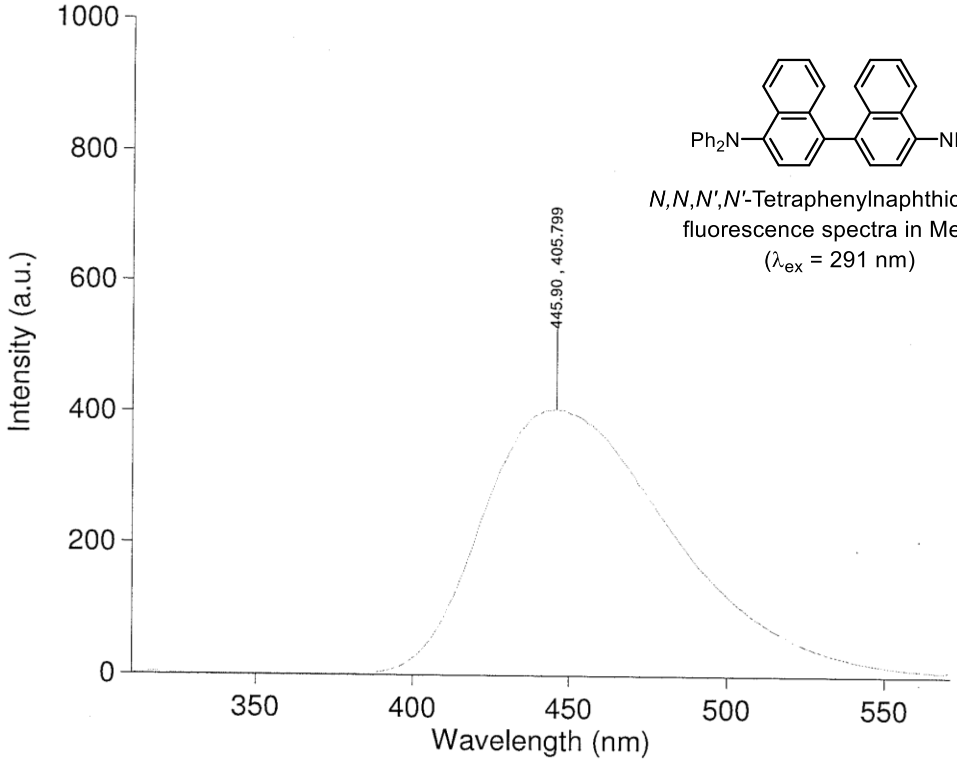

N,N,N′,N′-Tetraphenylnaphthidine (**4**)
fluorescence spectra in MeOH
(λ_{ex} = 291 nm)

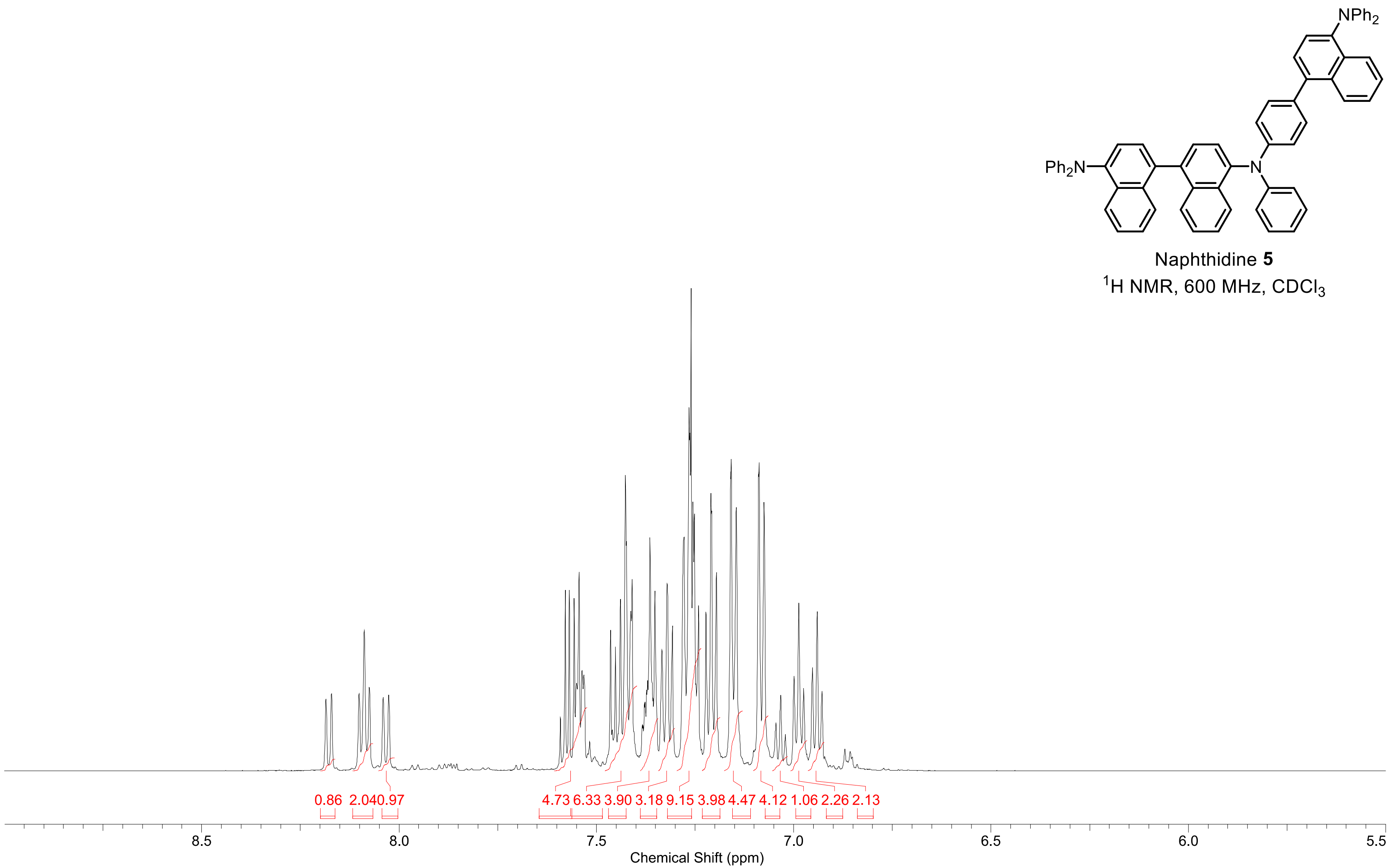

NPh2
Ph2N
N
Naphthidine 5
1H NMR, 600 MHz, CDCl3
0.86
2.04
0.97
4.73
6.33
3.90
3.18
9.15
3.98
4.47
4.12
1.06
2.26
2.13
8.5
8.0
7.5
7.0
6.5
6.0
5.5
Chemical Shift (ppm)

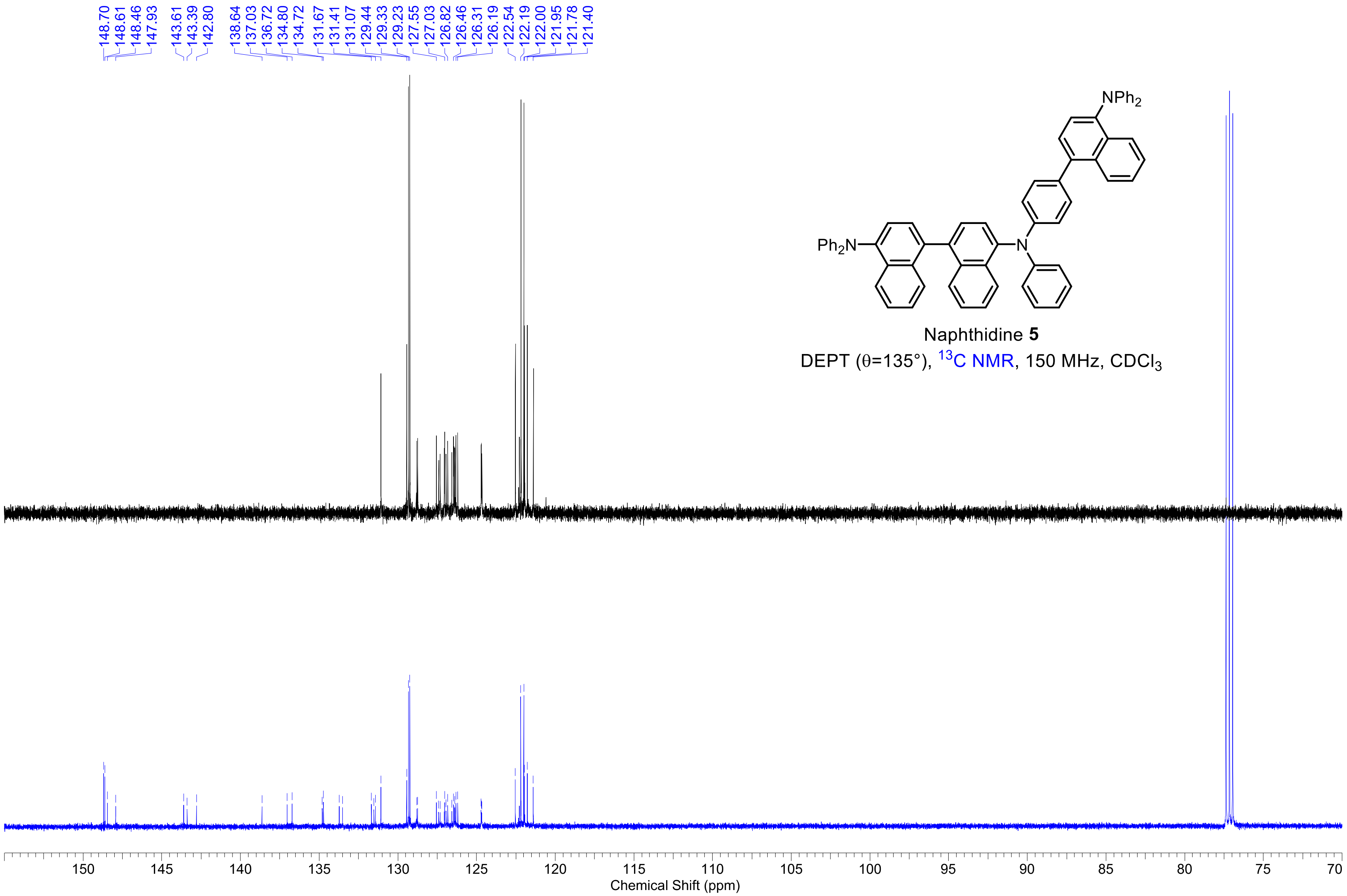
148.70
148.61
148.46
147.93
143.61
143.39
142.80
138.64
137.03
136.72
134.80
134.72
131.67
131.41
131.07
129.44
129.33
129.23
127.55
127.03
126.82
126.46
126.31
126.19
122.54
122.19
122.00
121.95
121.78
121.40
NPh2
Ph2N
N
Naphthidine 5
DEPT (θ=135°), 13C NMR, 150 MHz, CDCl3
150
145
140
135
130
125
120
115
110
105
100
95
90
85
80
75
70
Chemical Shift (ppm)

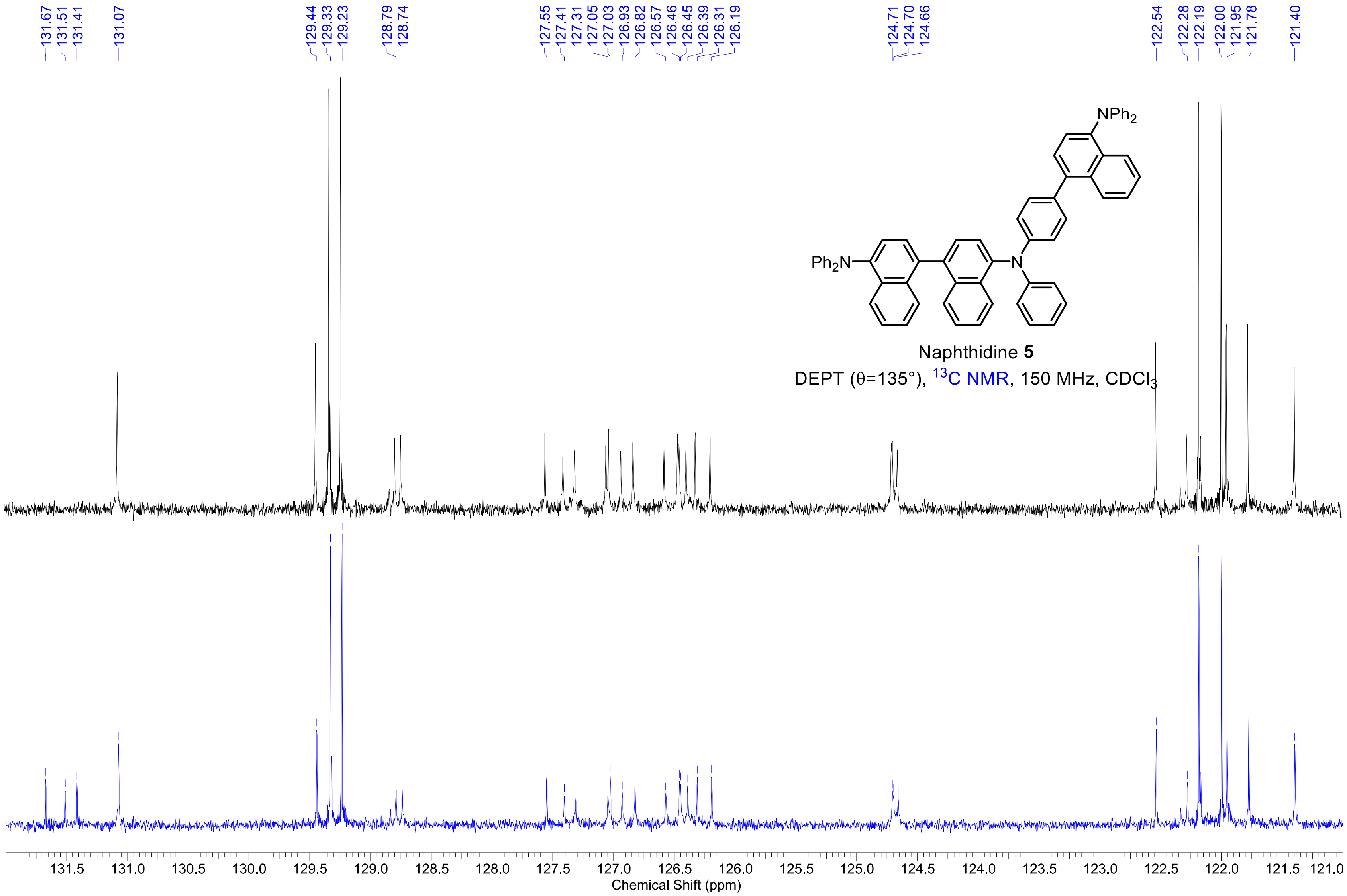

131.67
131.51
131.41
131.07
129.44
129.33
129.23
128.79
128.74
127.55
127.41
127.31
127.05
127.03
126.93
126.82
126.57
126.46
126.45
126.39
126.31
126.19
124.71
124.70
124.66
122.54
122.28
122.19
122.00
121.95
121.78
121.40
NPh2
Ph2N
N
Naphthidine 5
DEPT (θ=135°), 13C NMR, 150 MHz, CDCl3
131.5
131.0
130.5
130.0
129.5
129.0
128.5
128.0
127.5
127.0
126.5
126.0
125.5
125.0
124.5
124.0
123.5
123.0
122.5
122.0
121.5
121.0
Chemical Shift (ppm)

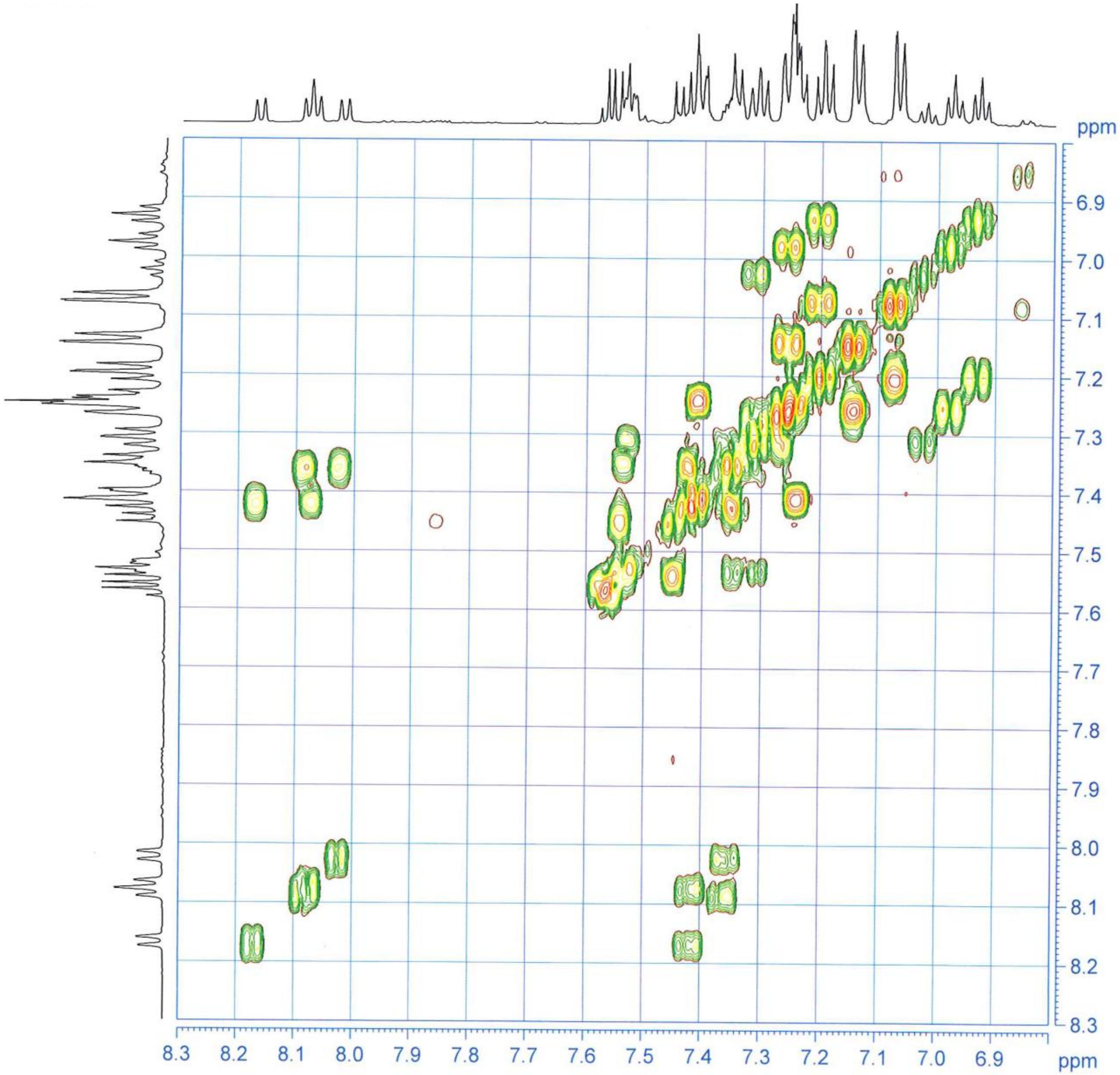

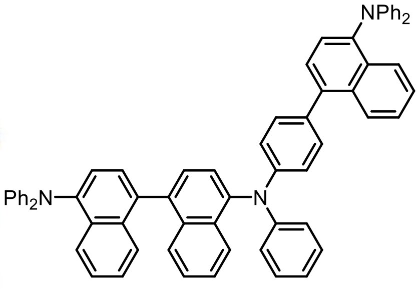

Naphthidine **5**
COSY, $CDCl_3$

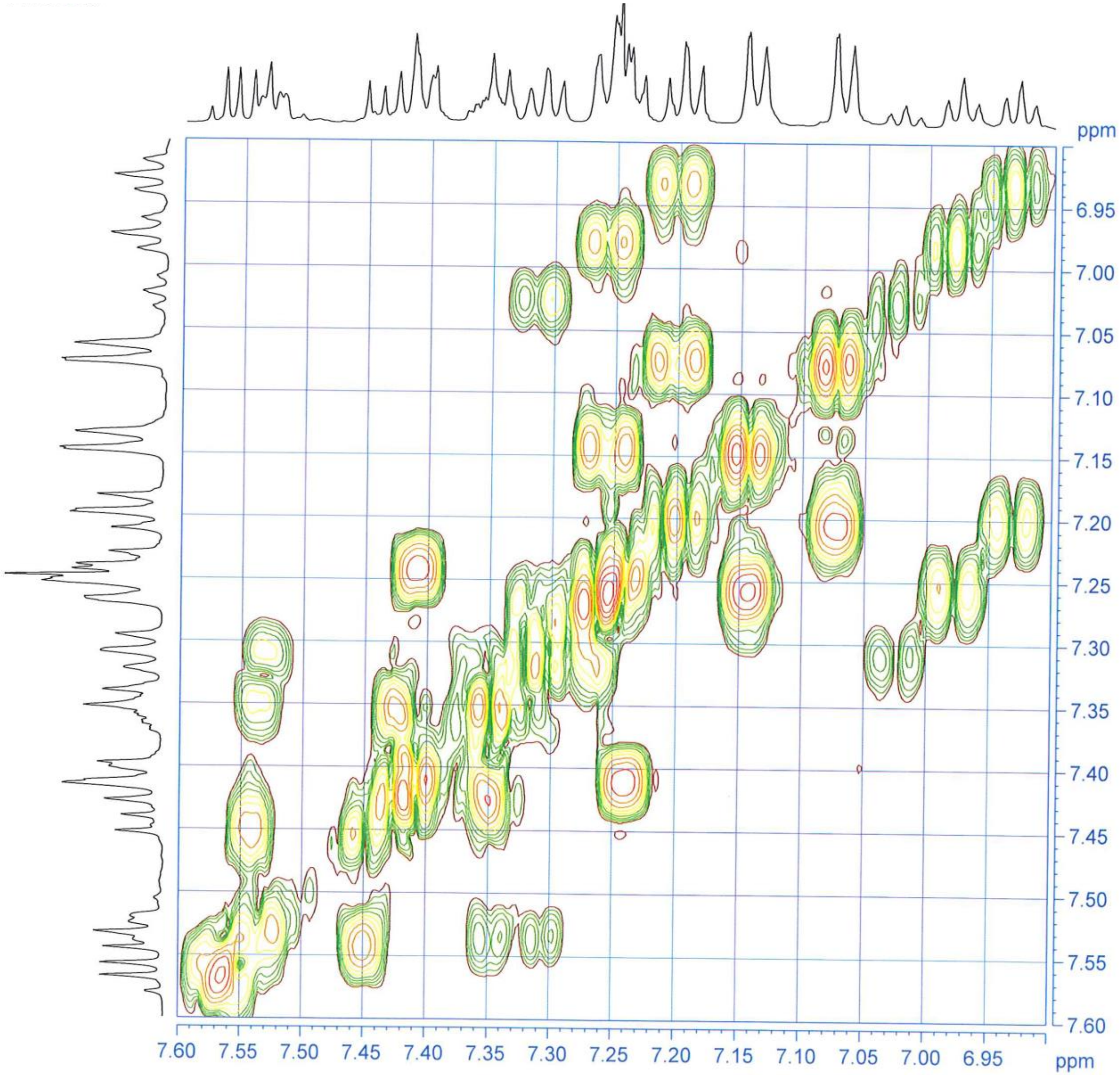

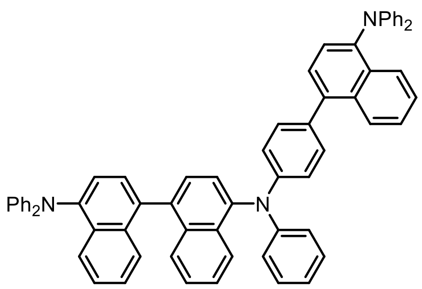

Naphthidine **5**
COSY, $CDCl_3$

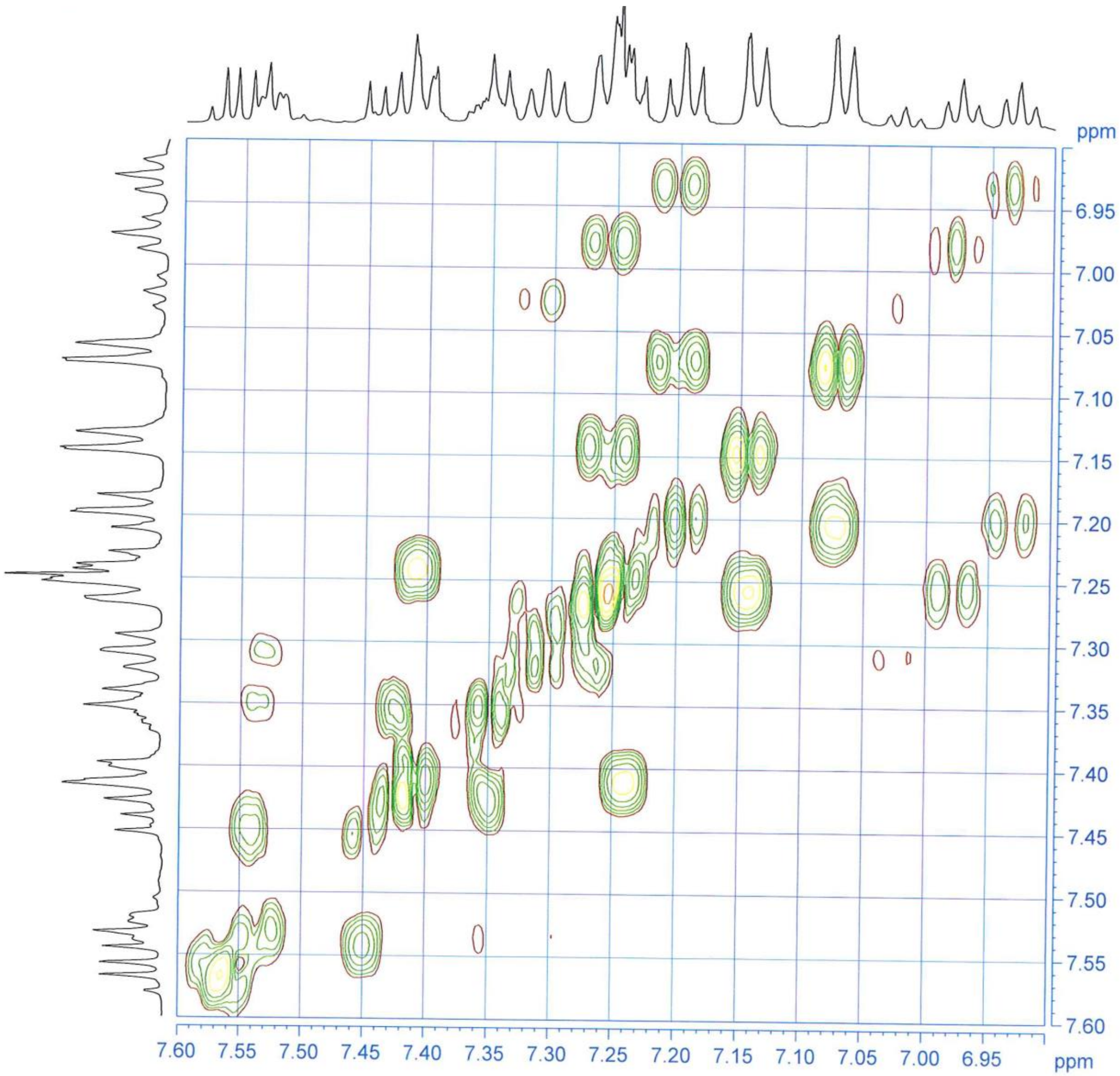

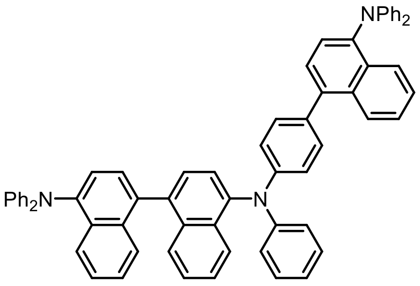

Naphthidine **5**
COSY, $CDCl_3$

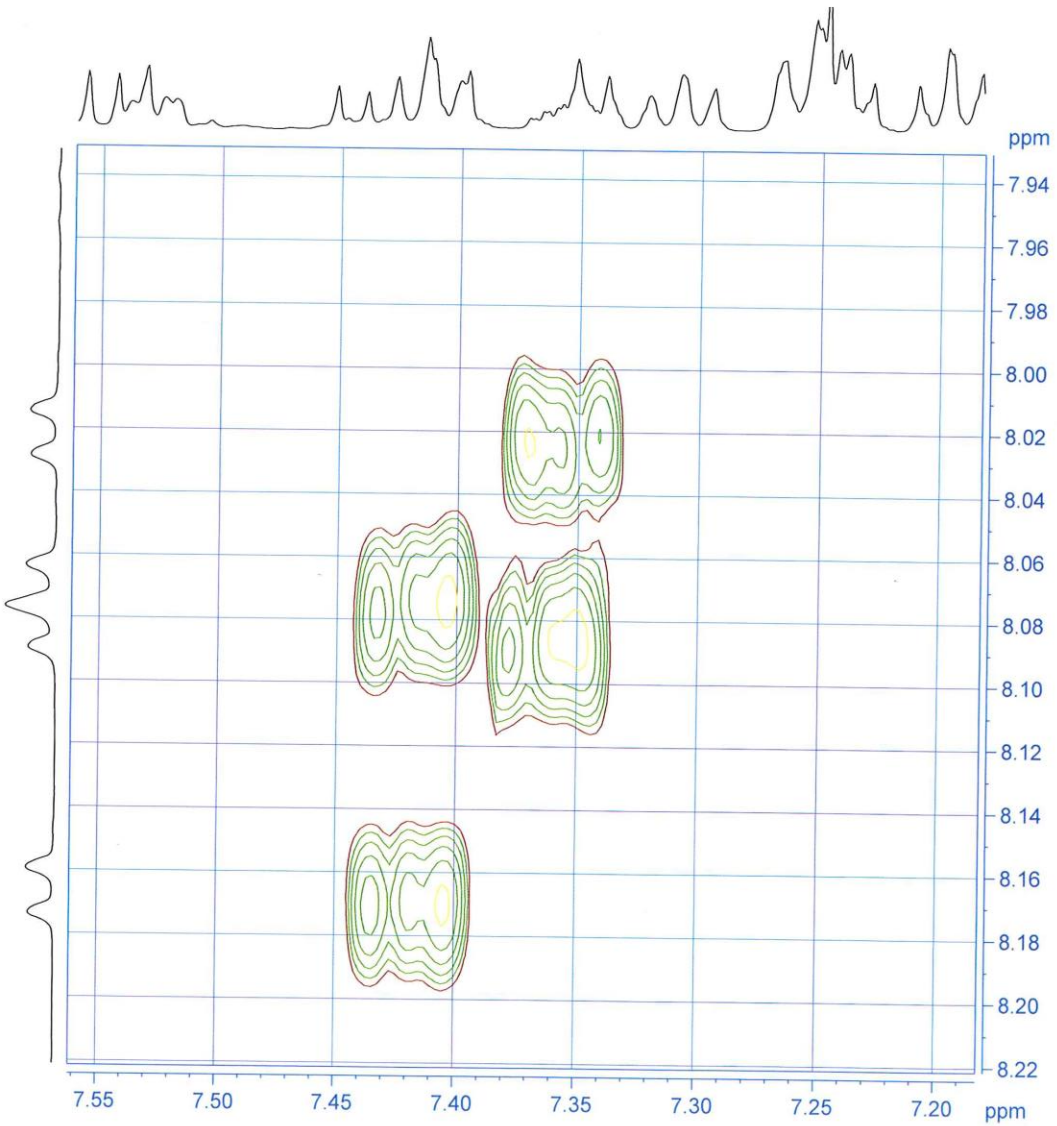

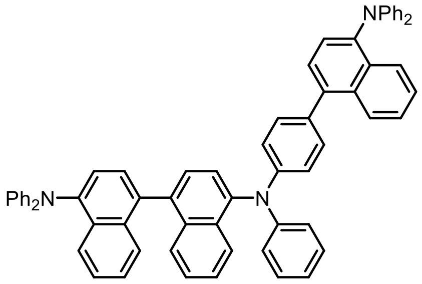

Naphthidine **5**
COSY, $CDCl_3$

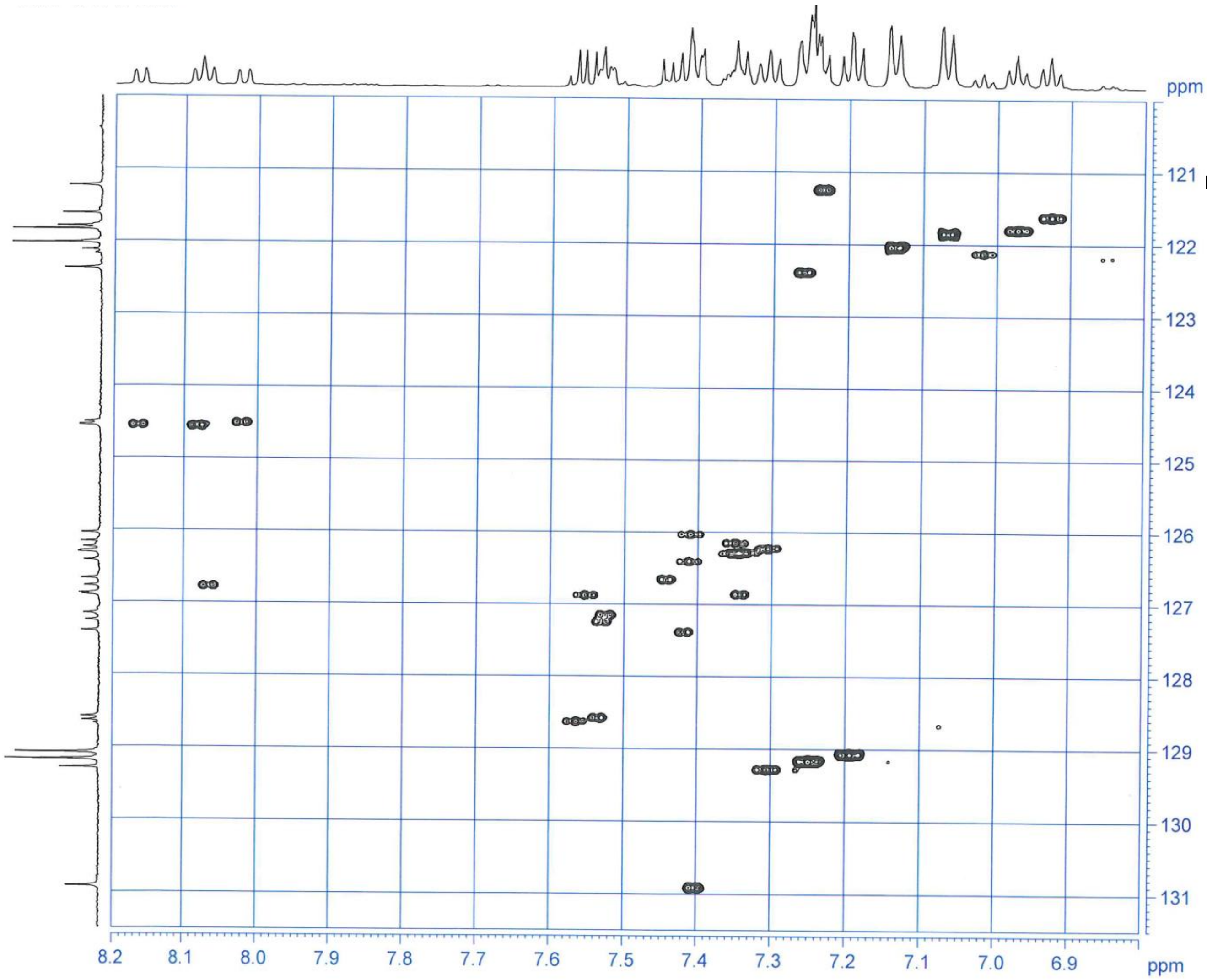

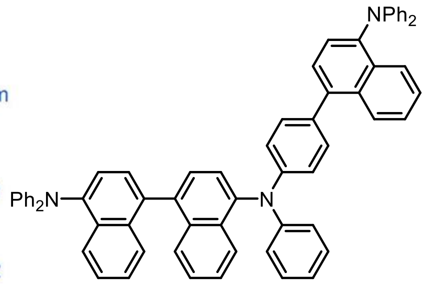

Naphthidine **5**
HSQC, $CDCl_3$

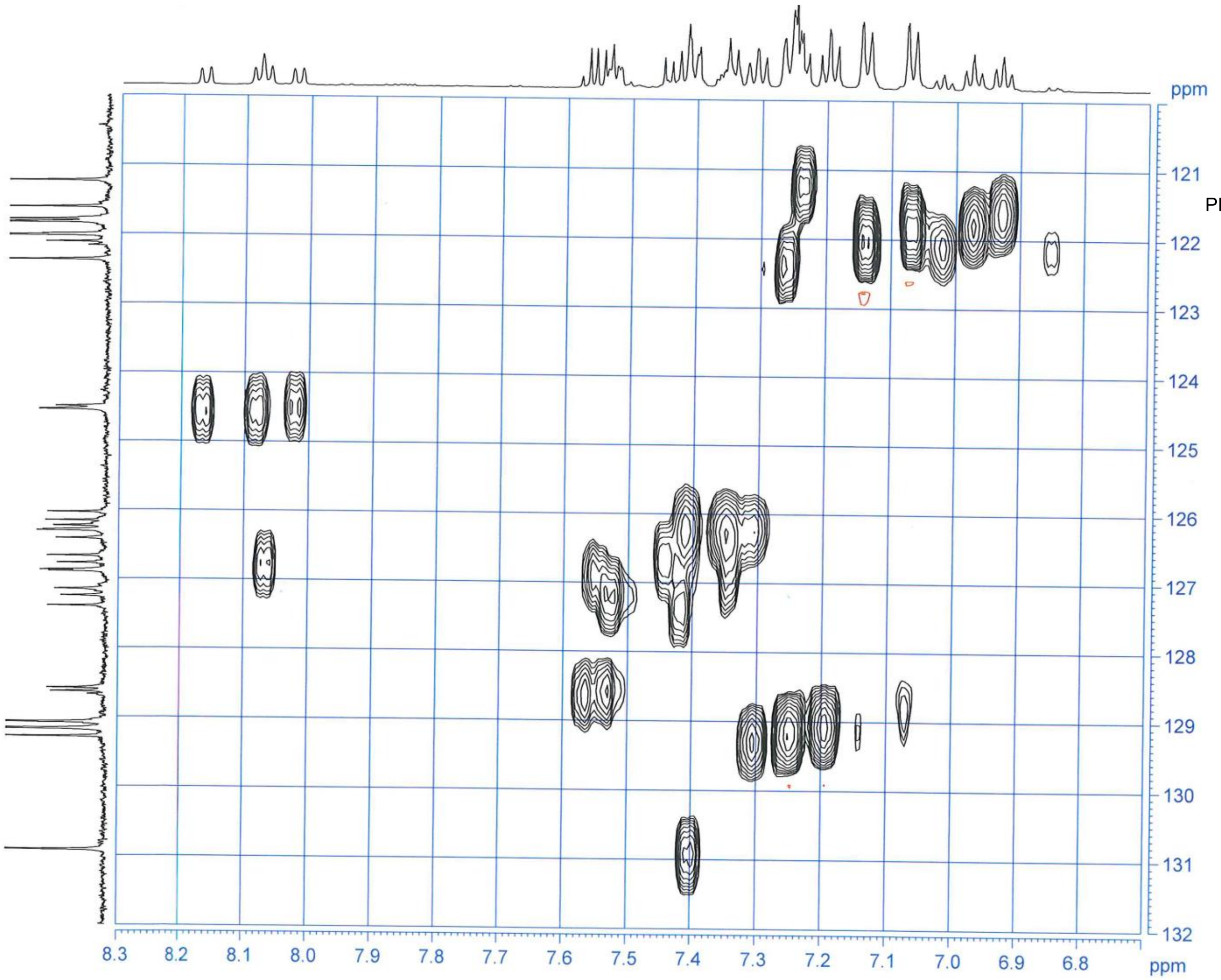

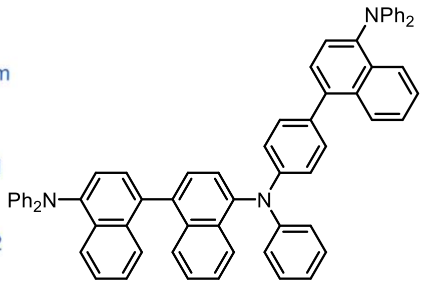

Naphthidine **5**
HSQC, $CDCl_3$

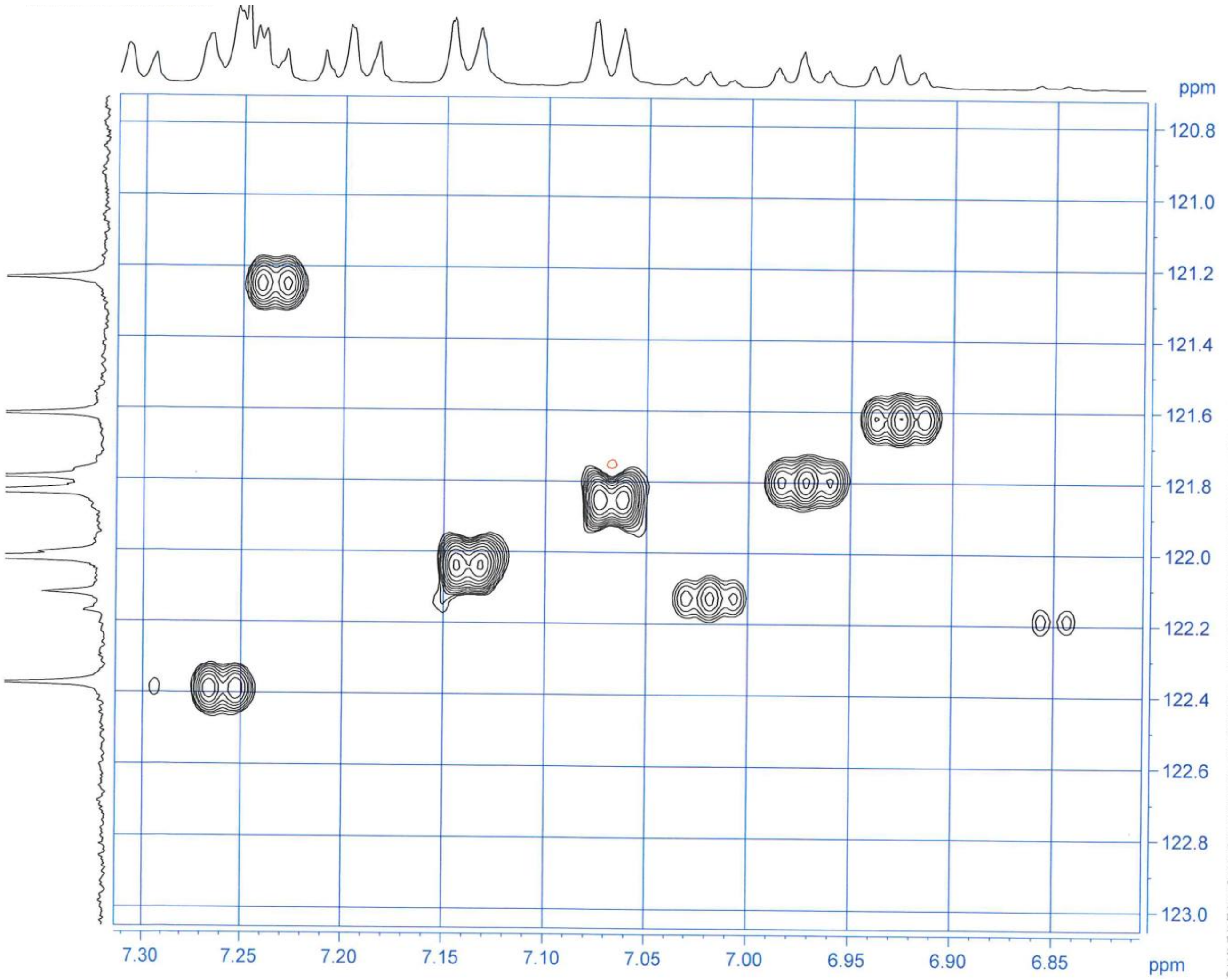

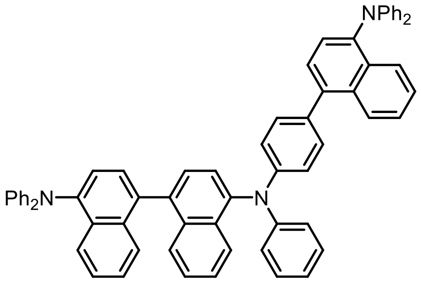

Naphthidine **5**
HSQC, $CDCl_3$

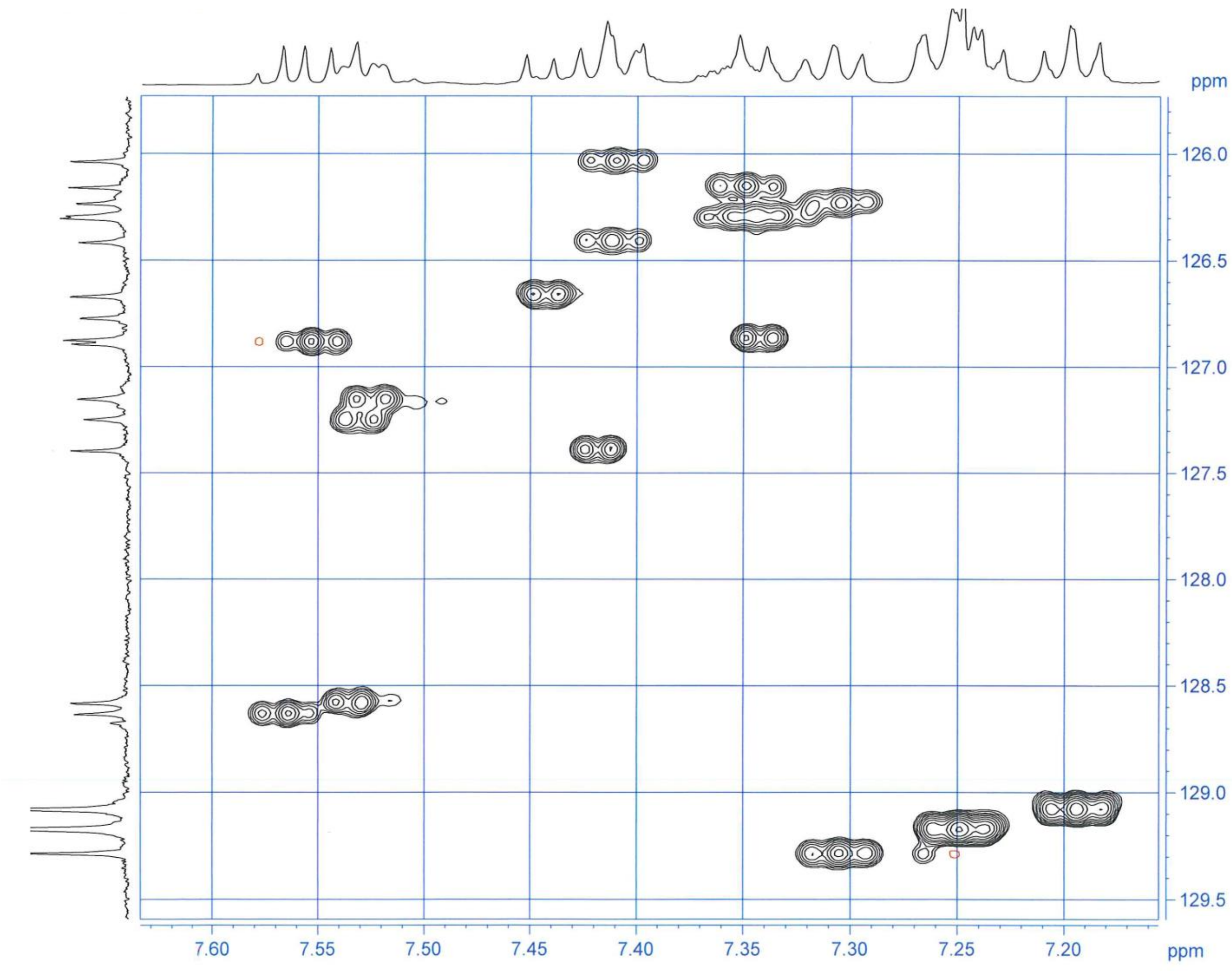

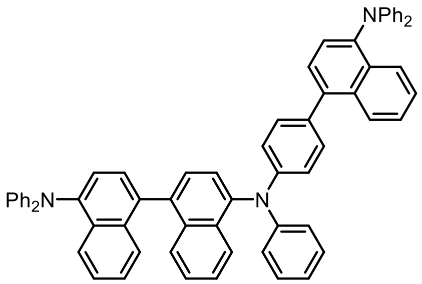

Naphthidine **5**
HSQC, $CDCl_3$

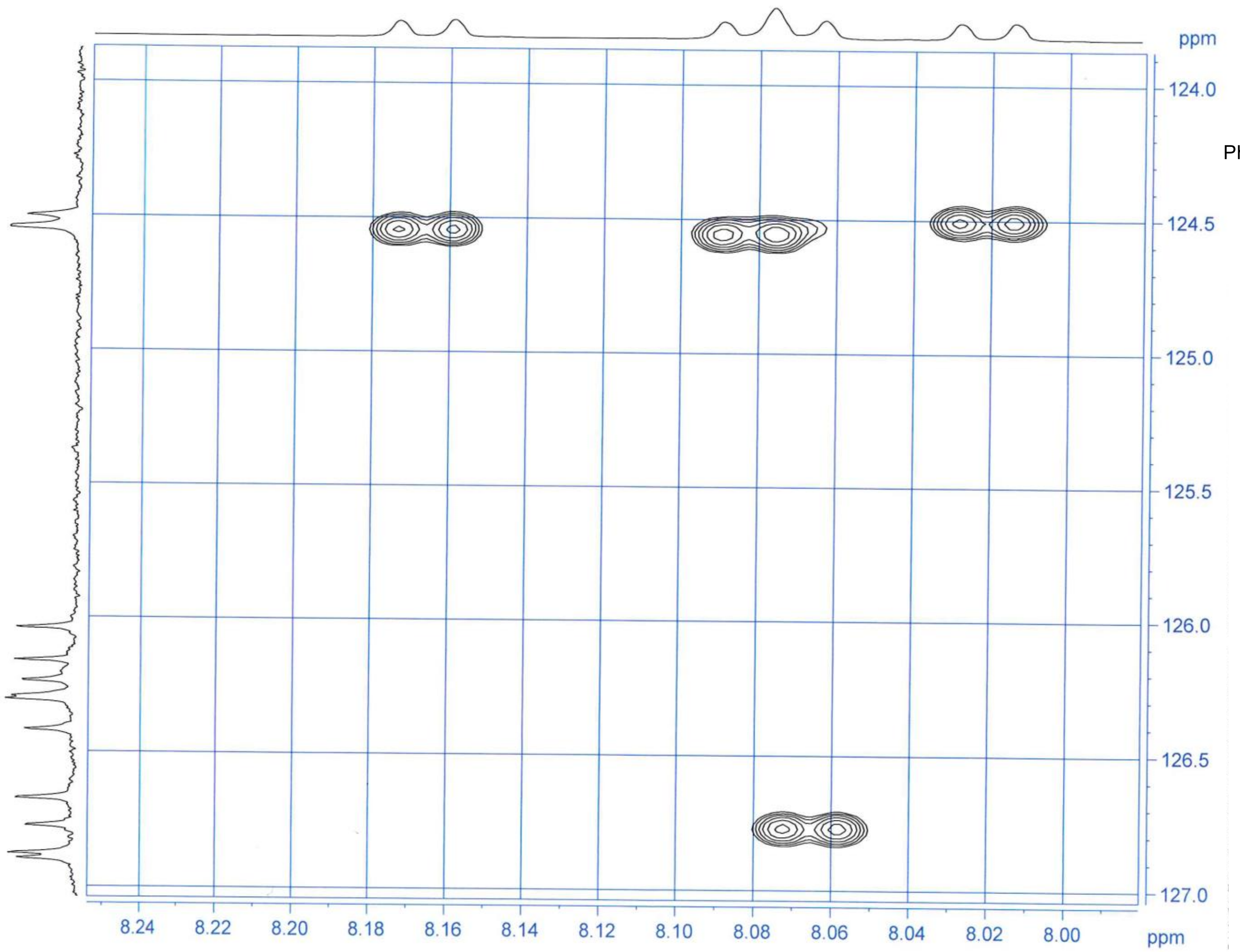

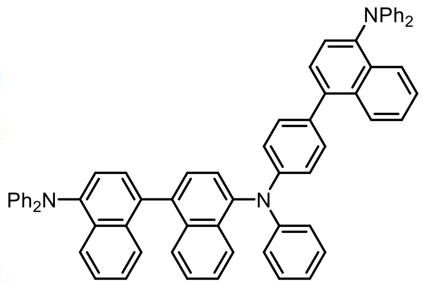

Naphthidine **5**
HSQC, $CDCl_3$

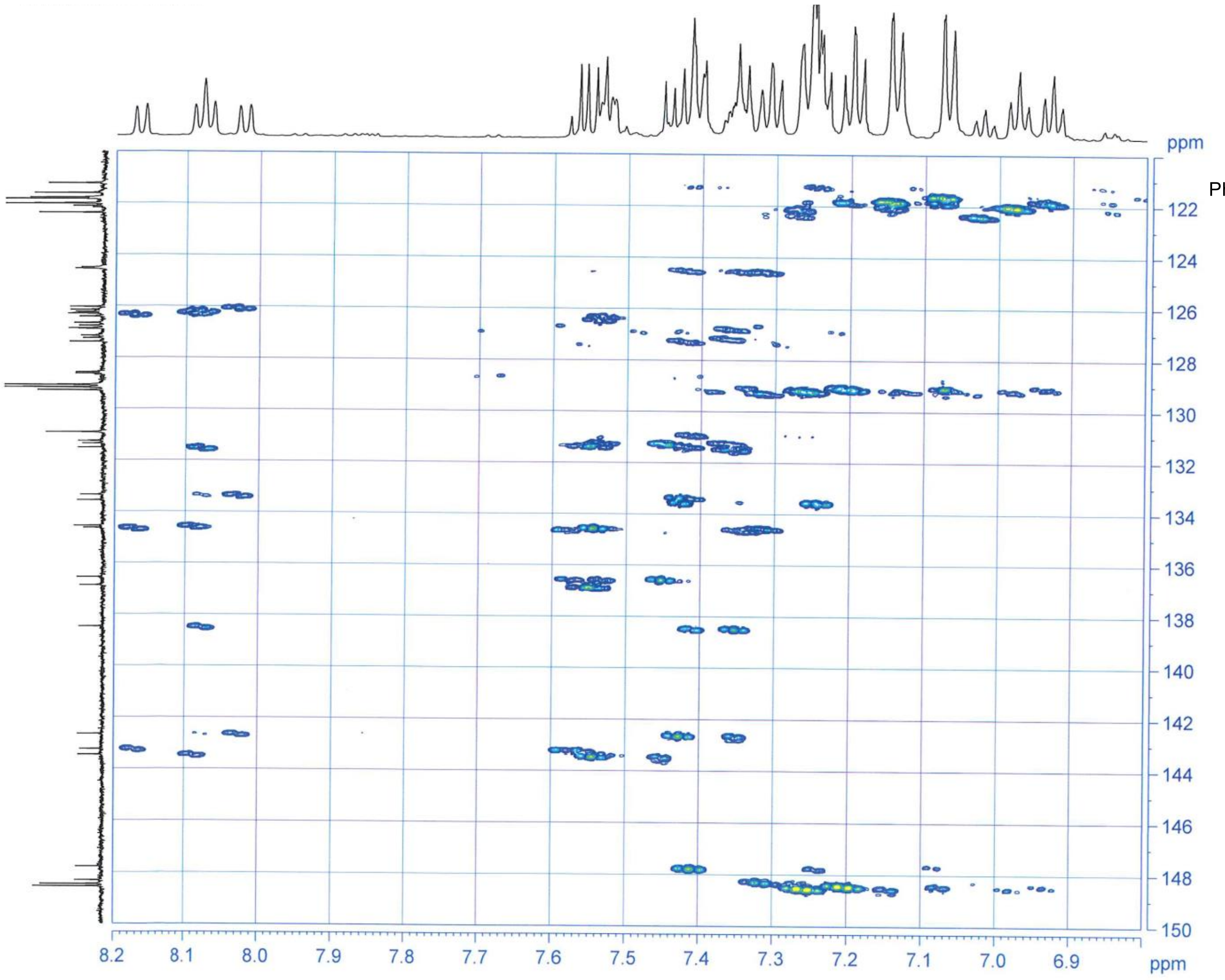

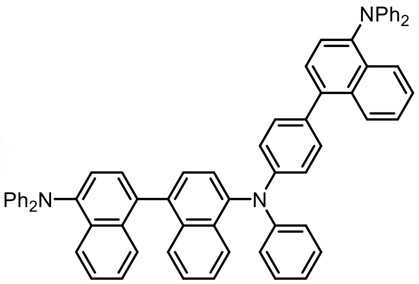

Naphthidine **5**
HMBC, $CDCl_3$

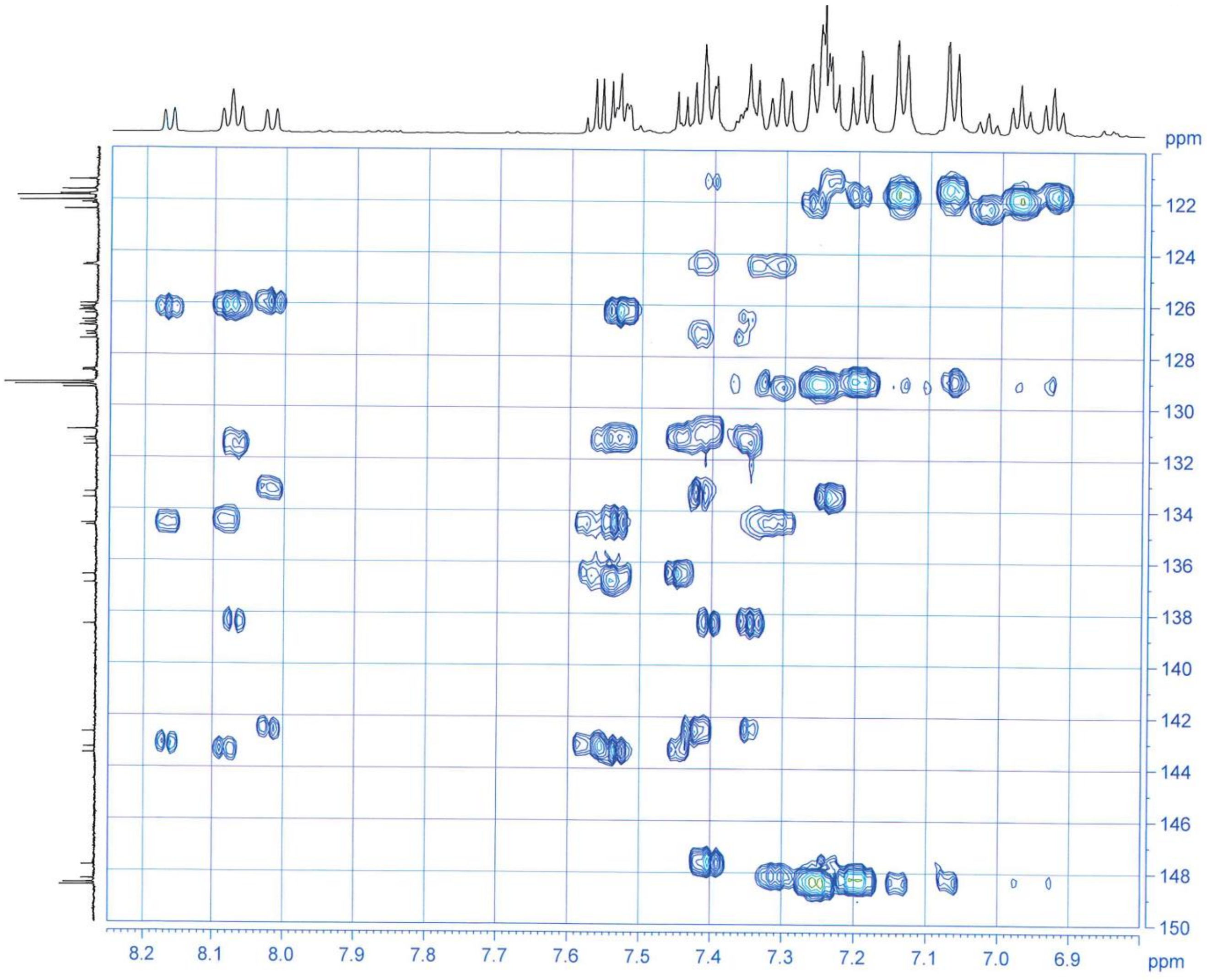

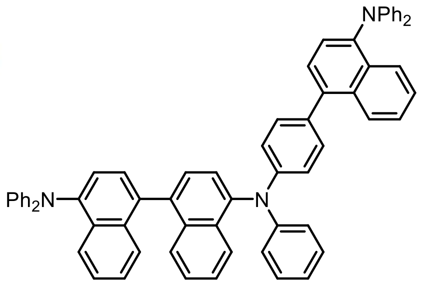

Naphthidine **5**

HMBC, $CDCl_3$

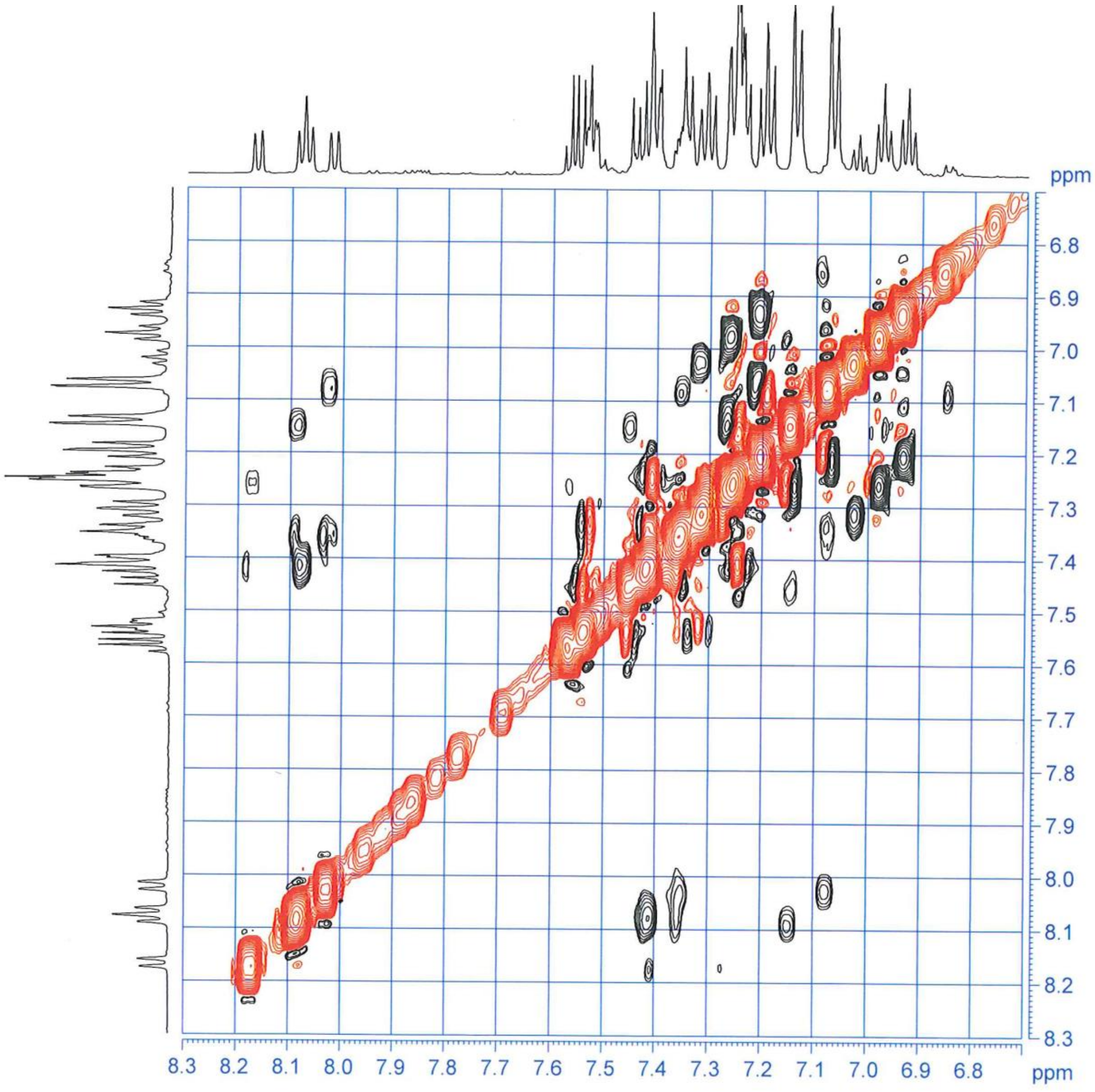

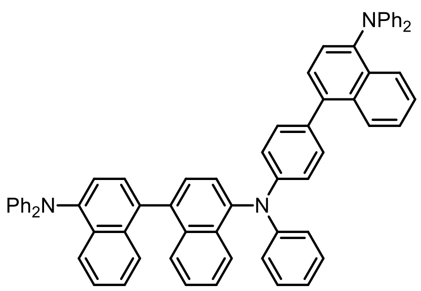

Naphthidine **5**
NOESY, $CDCl_3$

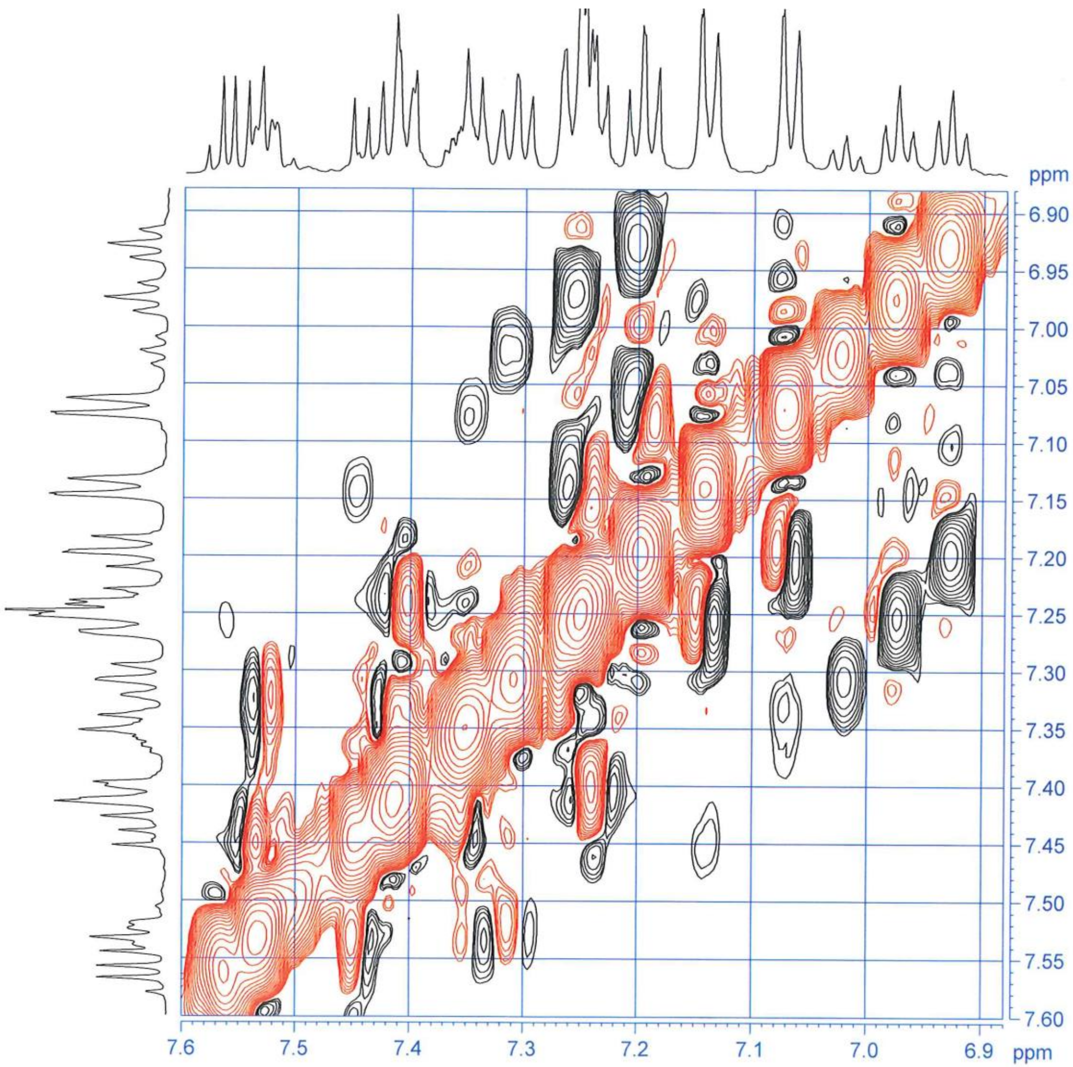

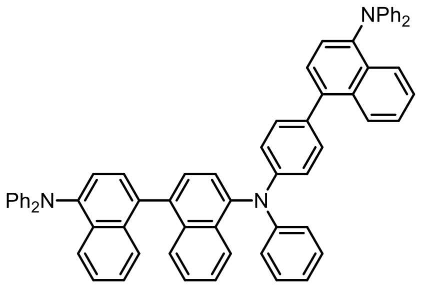

Naphthidine **5**
NOESY, $CDCl_3$

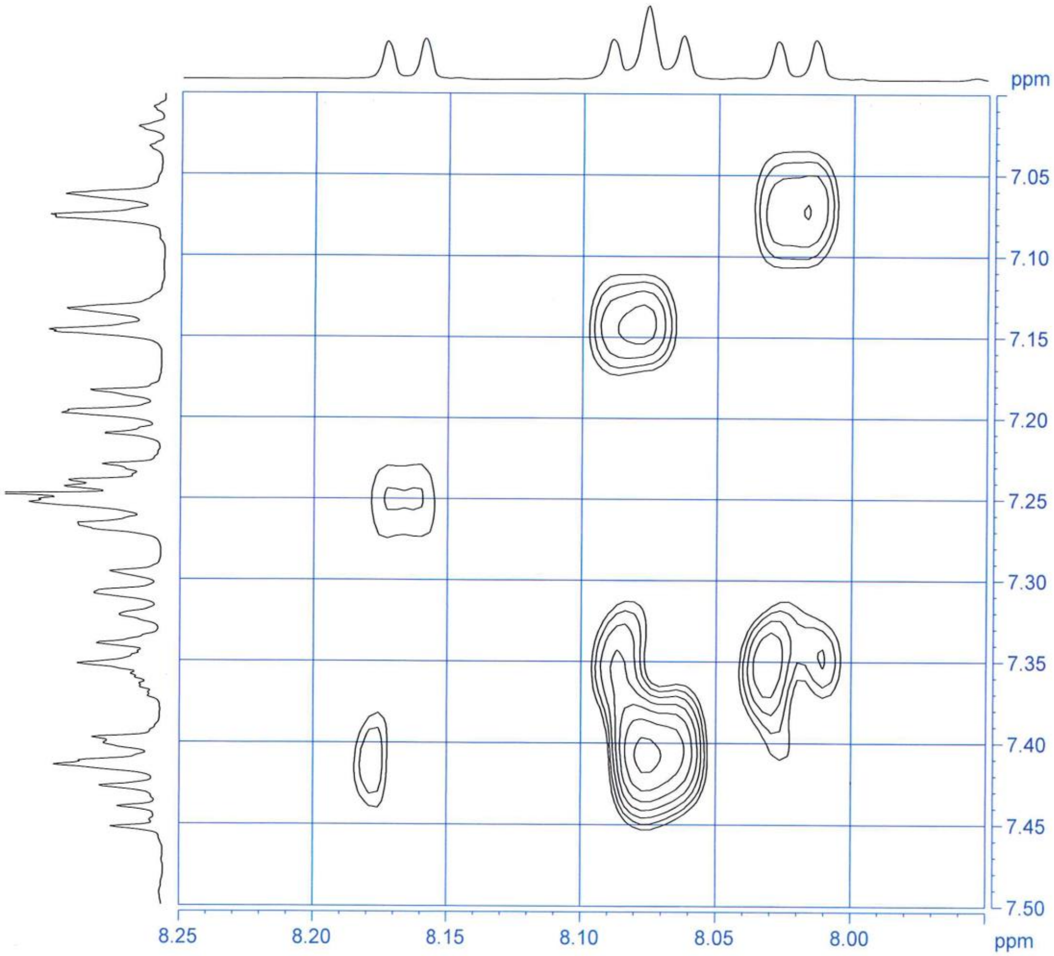

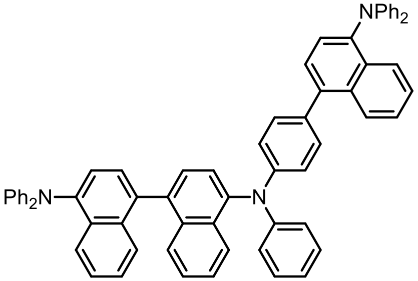

Naphthidine **5**
NOESY, $CDCl_3$

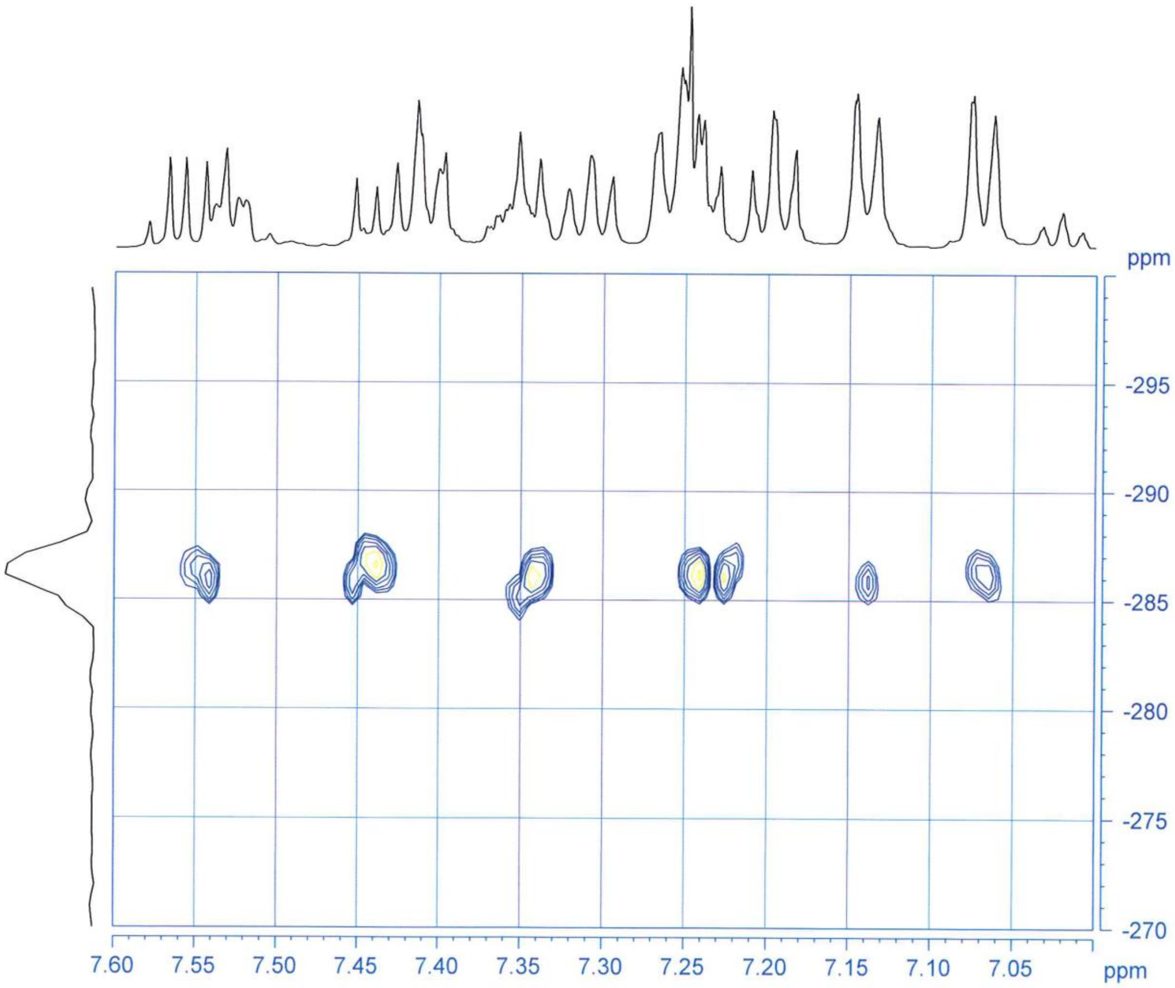

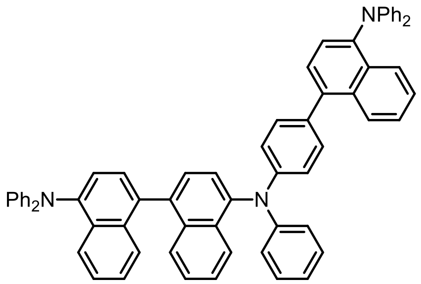

Naphthidine **5**

^{1}H/ ^{15}N HMBC, 600/ 61 MHz, $CDCl_3$

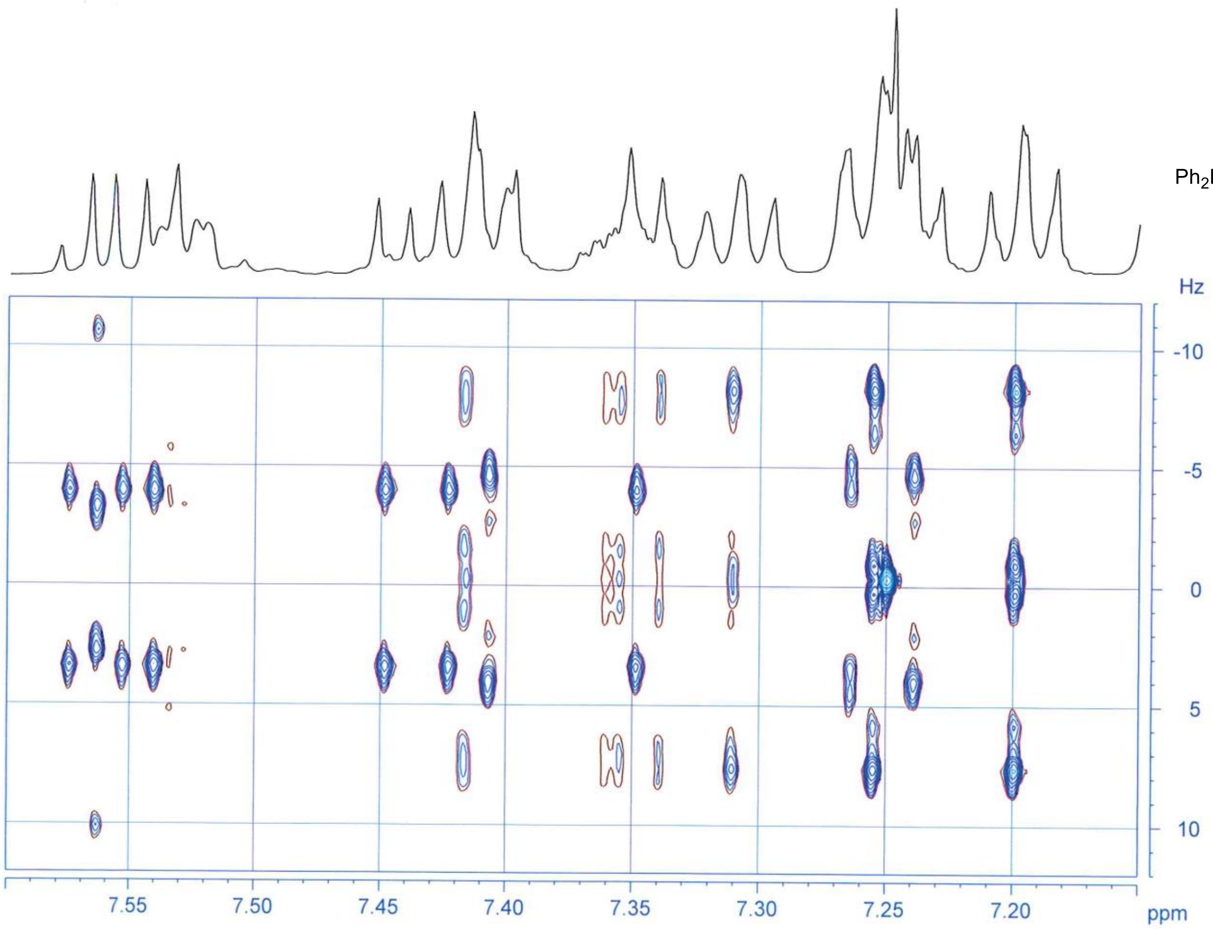

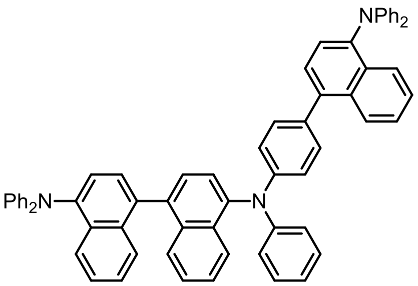

Naphthidine **5**
JRES, $CDCl_3$

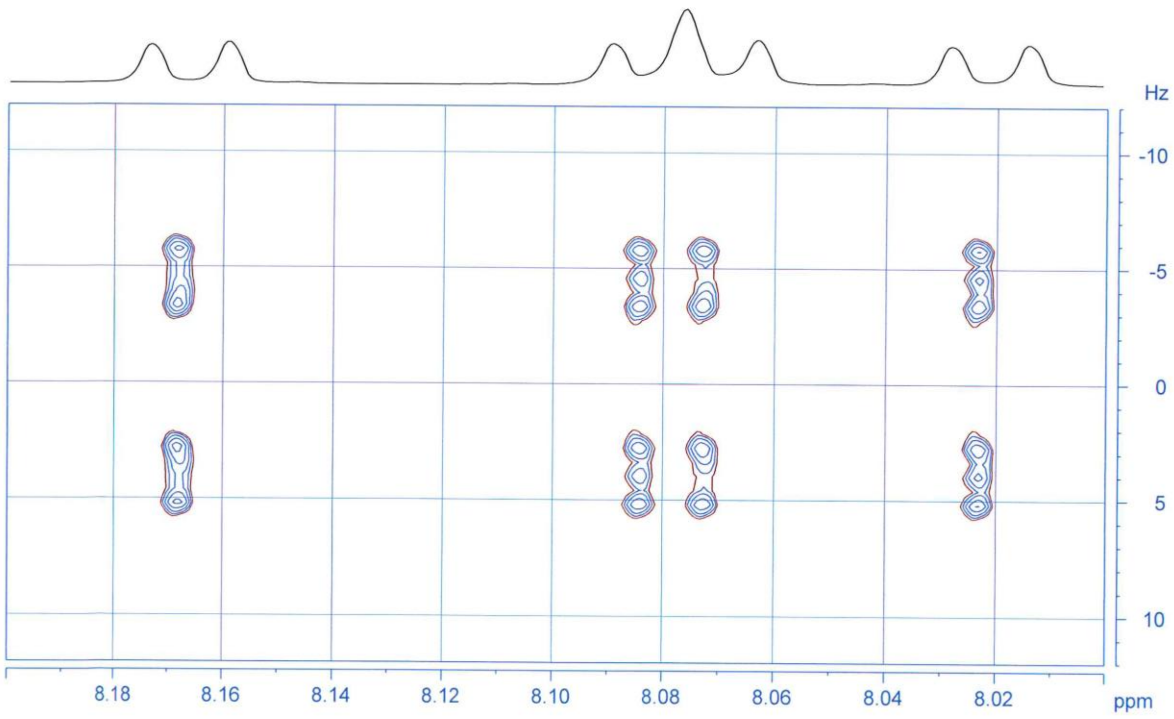

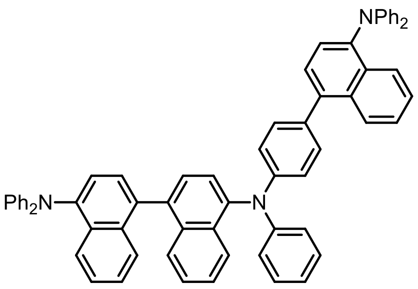

Naphthidine **5**
JRES, $CDCl_3$

Table S2. ^{13}C NMR data (150 MHz) of naphthidine **5** in $CDCl_3$.

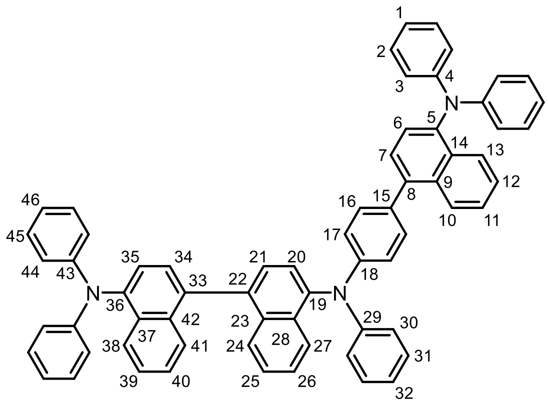

5

C	δc [a]
1	121.78 CH
2	129.23 CH
3	122.00 CH
4	148.61 C
5	142.80 C
6	127.55 CH
7	127.05 CH
8	138.64 C
9	133.72 C
10	126.93 CH
11	126.19 CH
12	126.31 CH
13	124.66 CH
14	131.67 C
15	133.51 C
16	131.07 CH
17	121.40 CH
18	147.93 C
19	143.39 C
20	127.03 CH
21	128.79 CH
22	137.03 C
23	134.80 C
24	127.41 CH
25	126.46 CH
26	126.57 CH
27	124.70 CH
28	131.51 C
29	148.46 C
30	122.54 CH
31	129.44 CH
32	122.28 CH
33	136.72 C
34	128.74 CH
35	126.82 CH
36	143.61 C
37	131.41 C
38	124.71 CH
39	126.45 CH
40	126.39 CH
41	127.31 CH
42	134.72 C
43	148.70 C
44	122.19 CH
45	129.33 CH
46	121.95 CH

[a] Number of attached protons as determined by the DEPT experiment (θ = 135°).

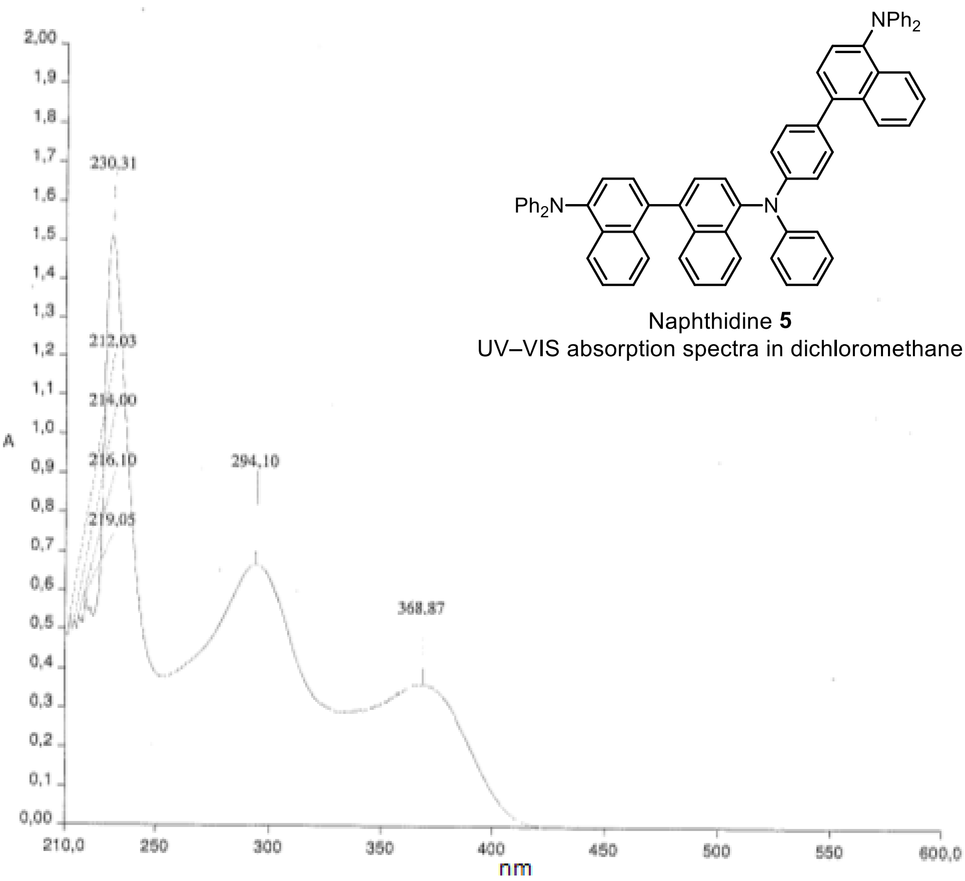

Naphthidine **5**
UV–VIS absorption spectra in dichloromethane

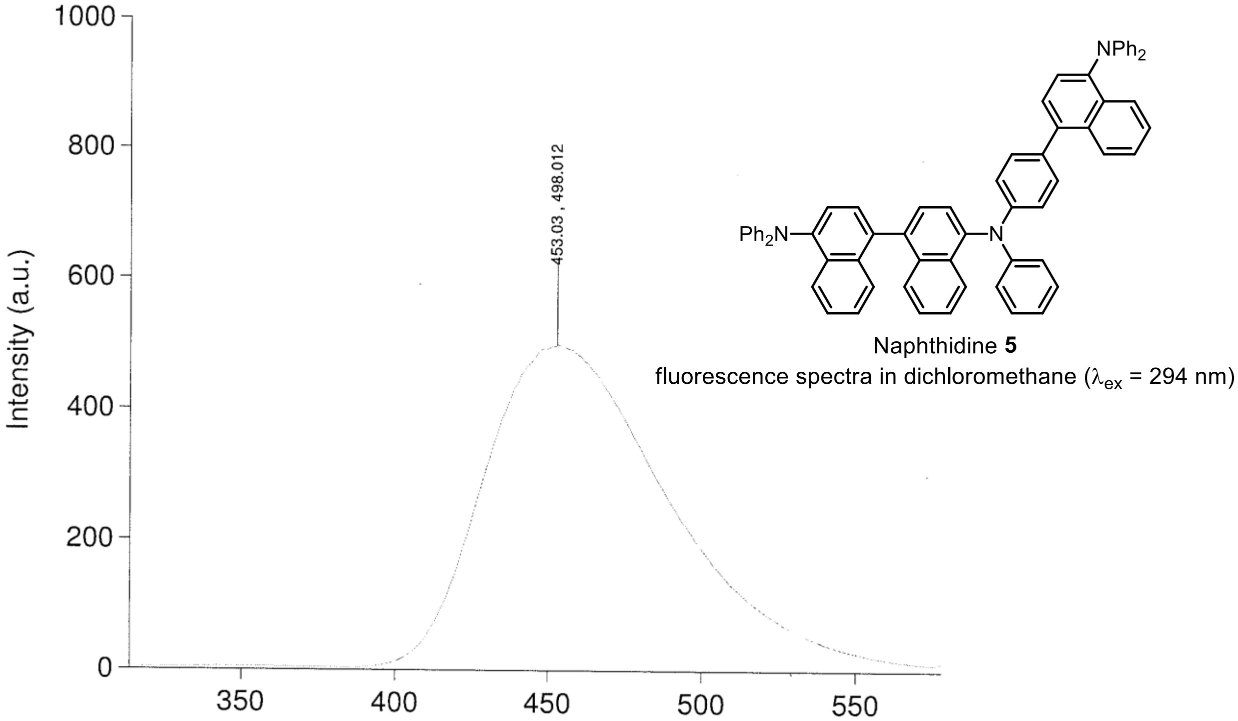

Naphthidine **5**
fluorescence spectra in dichloromethane (λ_{ex} = 294 nm)

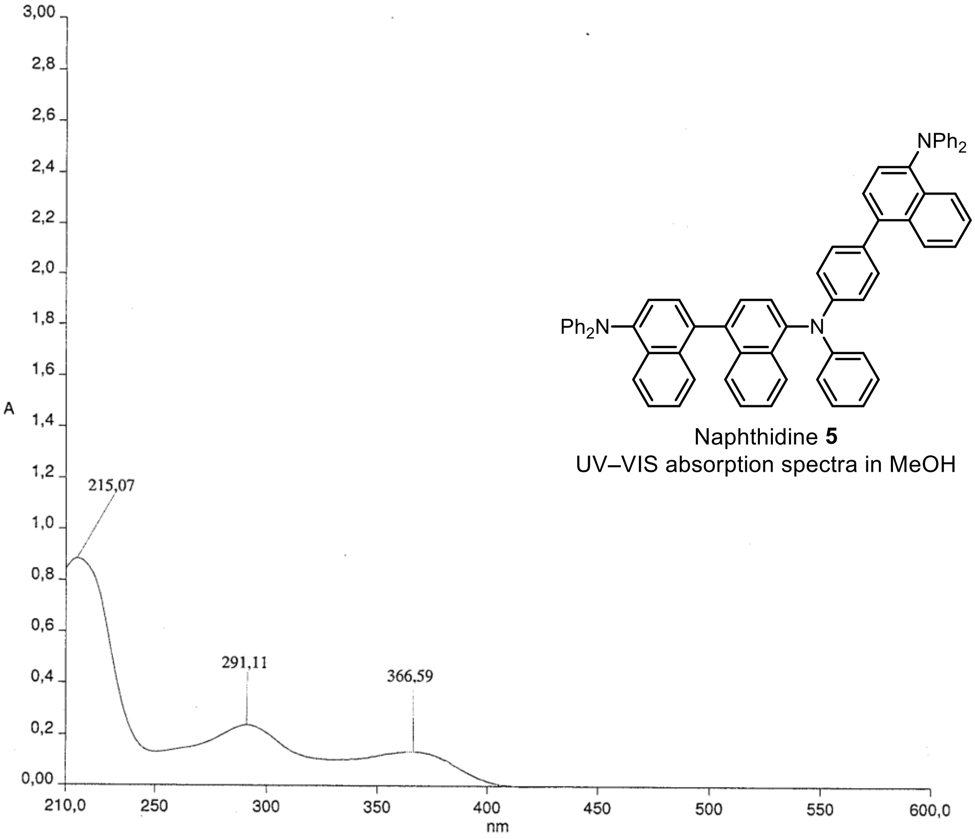

Naphthidine **5**
UV–VIS absorption spectra in MeOH

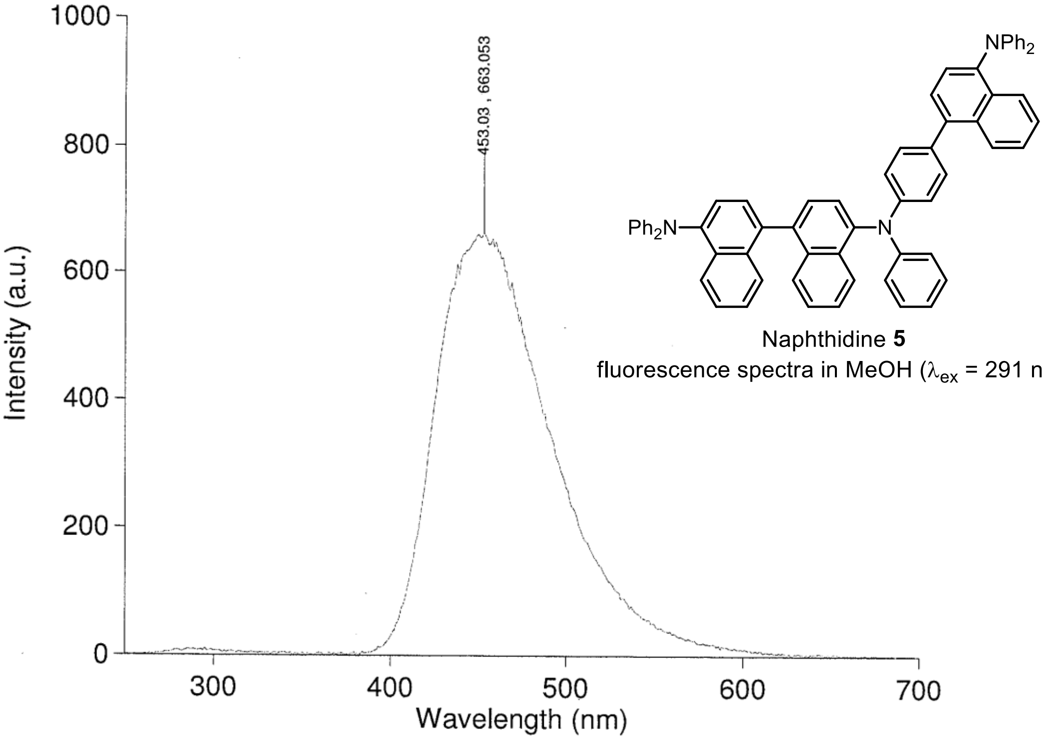

Naphthidine **5**
fluorescence spectra in MeOH (λ_{ex} = 291 nm)
